# How Have Alcohol Producers Changed the Alcohol Content of Their Products? A Descriptive Analysis of Reformulation in the Off‐Trade Alcohol Market in Great Britain, 2018–2025

**DOI:** 10.1111/dar.70197

**Published:** 2026-06-30

**Authors:** Colin Angus, Luke Wilson, Sarah Jackson, Nathan Critchlow, Grace Leeming, Jamie Brown, John Holmes

**Affiliations:** ^1^ Sheffield Addictions Research Group, School of Medicine and Population Health University of Sheffield, Regent Court Sheffield UK; ^2^ Department of Behavioural Science and Health University College London London UK; ^3^ Institute for Social Marketing and Health University of Stirling Stirling UK

**Keywords:** alcohol consumption, alcohol policy, public health

## Abstract

**Introduction:**

In August 2023, the United Kingdom introduced alcohol taxation reforms designed to encourage alcohol producers to lower the alcoholic strength of their products. This study aims to quantify the extent of reformulation of alcoholic drinks sold in the off‐trade in Great Britain between 2018 and 2025 and explore the role the tax reforms may have played.

**Methods:**

We used continuous longitudinal data on alcohol purchases from Worldpanel by Numerator's Take Home data to examine changes between 2018 and 2025 in the mean alcohol‐by‐volume (ABV) of all alcohol sold, identify specific product reformulations and examine how their timing related to the 2023 tax reforms. We also explored growth in the < 3.5% ABV beer market, for which tax rates were cut in the reforms.

**Results:**

The average ABV of all alcohol rose from 17.2% in late 2018 to 17.7% in June 2022, before falling to 16.7% in December 2025. We identified 557 reformulations, of which 50% were for wine and 17% were for beer. Reformulations increased substantially following the reforms, with the proportion of the beer market, measured in pure alcohol, sold below 3.5% ABV increasing from 1.1% in 2022 to 18.1% in 2025.

**Discussion and Conclusions:**

Our findings suggest that the 2023 UK alcohol tax reforms appear to have contributed to an increase in reformulations that reduced the strength of alcoholic drinks. In turn, these may have played a role in reductions in the overall ABV of alcoholic drinks purchases. Following the reforms, there was a large and immediate increase in the market share of lower‐strength beers.

## Key Point

- Reformulations of alcoholic products have been relatively common in Great Britain between 2018 and 2025, with almost 5% of all products in the market reformulating at some point during this period.
- The majority of reformulations (76%) were reductions in the alcoholic strength of products.
- Reforms to the alcohol tax system in 2023 appear to have led to a substantial increase in reformulations, particularly for lower‐strength beer.
- Tax incentives can play an important role in encouraging the reformulation of alcohol products.

## 1 Introduction

Alcohol consumption is a leading risk factor for ill health and premature mortality, both globally and in Great Britain, particularly among working age adults [1, 2]. In 2023, 10,115 people died from conditions that are wholly caused by alcohol, the highest number on record, representing a 40.3% increase since 2019 [3]. Levels of alcohol harm are primarily driven by the volume of alcohol consumed, and effective interventions to reduce this include increasing alcohol prices or reducing its availability [4]. Another approach to achieving reductions in alcohol consumption is promoting the reformulation of products to reduce their alcoholic strength.

Promoting reformulation of harmful commodities has had limited success when enacted as a voluntary initiative where policymakers work in partnership with manufacturers [5, 6]. However, it has been more successful when governments have designed regulatory measures (e.g., taxes) to provide companies with economic incentives to improve the nutritional content of foods and non‐alcoholic drinks, with the UK Soft Drinks Industry Levy being a notable example [7, 8].

In October 2021, the UK Government announced reforms to its alcohol duty system, which it then implemented in August 2023 for most products and in February 2025 for wine (see Box 1 for interim measures for wine). The reforms introduced new incentives for reformulation of alcoholic drinks and altered pre‐existing ones via three main mechanisms [9]. First, they changed the basis for alcohol taxes on both wine and cider so that all alcoholic drinks are now taxed on the basis of their alcohol content—in other words, producers pay less tax on lower strength drinks. Second, the reforms raised the threshold below which beers paid a lower duty rate from 2.8% to 3.5% alcohol‐by‐volume (ABV) and applied this to all products with the explicit aim of encouraging reformulation [10]. Third, and conversely, the threshold above which beers paid a higher duty rate increased from 7.5% to 8.5% and was again applied to all products.

Box 1 summarises the potential incentives for reformulation under the new duty system for different types of alcohol, while Figures A1–A6 in the Supporting Information illustrate the reforms visually alongside a more detailed discussion of these incentives. At the same time as the reforms were implemented, a 10.1% inflation‐linked increase was applied to all alcohol duties, the largest cash‐terms increase for several decades [11]. This did not change the incentive structures within the duty system but did affect the pressure on manufacturers to engage with those structures by raising their tax burden.

BOX 1 **Potential incentives for alcohol product reformulation following the alcohol duty reforms in August 2023.**

**Table:** 

**Beer** Already taxed on the basis of alcohol content, so no new incentive at most alcohol‐by‐volume (ABV) levels. Raising the threshold for the lower‐strength duty band from 2.8% to 3.5% sought to encourage brewers to reformulate products to below 3.5%. Raising the higher‐strength duty threshold from 7.5% to 8.5% created a new incentive for > 8.5% beers to reduce their strength to 7.5–8.4%, and removed a potential disincentive for producers to reformulate from < 7.5% into this higher range. **Cider** Duty levied based on alcohol content for the first time, so producers have a new incentive to reformulate across all ABV levels. Lower‐strength rate at < 3.5% also applies to cider, but the reduction in duty for a reduction in ABV is much smaller than for beer, so the incentive is weaker. **Wine** Duty levied based on alcohol content for the first time. However, interim measures meant that all wine between 11.5% and 14.5% was temporarily taxed as if it were 12.5%. This retained the previous lack of incentive to reformulate within this range. The interim measures did create a temporary threshold with a lower duty rate for wines below 11.5% ABV, meaning there was a new incentive for producers to reformulate to below this threshold. **Spirits** Already taxed on the basis of alcohol content. The new system introduced a lower duty rate for products below 22%, giving some lower‐strength spirits (e.g., liqueurs) an incentive to reformulate to below this threshold. **‘Ready‐To‐Drinks’―pre‐mixed wine‐ or spirit‐based drinks** Already taxed on the basis of alcohol content, but now taxed at a lower rate under the new system. Lower‐strength duty band incentivises reformulation to below 3.5% ABV and this incentive is stronger than for beer and cider because the duty rate for products at or above 3.5% is higher for Ready‐To‐Drinks.

These reforms are not the only major changes affecting alcohol manufacturers in recent years, and several of the others also affect incentives for reformulation. The introduction of Minimum Unit Pricing in Scotland in May 2018, followed by Wales in March 2020, led to substantial increases in price for some high‐strength, low‐price products, particularly cider [12, 13]. However, the incentive to reformulate here is offset by businesses rather than government accruing the additional revenue from selling alcohol at higher prices. High levels of inflation between late 2021 and early 2024 also led to a ‘cost‐of‐living crisis’ [14], intensifying financial pressure on consumers, particularly those on lower incomes [15, 16] while increasing costs for alcohol producers and retailers. At the same time, consumer trends around alcohol have been changing, with the continued growth of the alcohol‐free and low‐alcohol drinks market [17, 18] and a general trend towards lower‐strength drinks [19] that have coincided with a fall in overall alcohol sales since early 2022 [20]. More broadly, the onset of the COVID‐19 pandemic in March 2020 led to a large‐scale shift in purchasing from the on‐trade (pubs, bars, restaurants, etc.) to the off‐trade (convenience stores, supermarkets, etc.) and saw significant changes in individual‐level alcohol consumption [21] that may affect economic pressures facing manufacturers.

While reformulation has the potential to improve public health—if consumers drink the same volumes of alcoholic products at a lower ABV, they will consume less alcohol overall, reducing their health risks—there has been little research to date to quantify the scale of reformulation across the alcohol market or explore how the timing of reformulations may be linked to events such as the duty reforms. One previous study used data from a single UK retailer from 3 months before and after the duty reforms and found some evidence of reformulation, particularly for lower‐strength products [22]. Another used older data for beer only and found some evidence of reformulation between 2015 and 2018 [23].

In this study we build on these analyses using a large‐scale sample of data on off‐trade alcohol purchases in Great Britain (the nations of England, Scotland and Wales) between 2018 and 2025 to: (i) quantify how the mean ABV of alcohol sold has changed; (ii) identify reformulations of at least 0.1% ABV and the proportion of the market they affect; (iii) describe how the timing of these reformulations aligns with the 2023 alcohol duty reforms; and (iv) quantify changes in the magnitude of the sub‐3.5% ABV beer market. We focus on the sub‐3.5% beer market due to the explicit aim to increase reformulation around this threshold in the duty reforms, recent media interest in several major brand reformulations that crossed this threshold (e.g., [24, 25]) and the substantial proportion of the beer market that exists close to this threshold (see Supporting Information Figure A2).

## 2 Methods

This study was preregistered [26].

### 2.1 Data

This study uses data collected by the market research company Worldpanel by Numerator (formerly Kantar) through their Take Home panel from January 2018 to December 2025. Worldpanel is a continuous panel of approximately 30,000 households across Great Britain. The Worldpanel sample is recruited through stratified quota sampling, with quotas set for geographic region, household size, presence of children and age of main shopper. Socioeconomic group is not included in the sample targets but is part of the weightings used to ensure the survey population is representative of Great Britain. The same households provide longitudinal data over time, with continuous replenishment to replace households that leave the sample and ensure the panel remains representative.

Participating households record the price and full product details of all food and drink products brought into the home. The data used in this study covers all purchases of alcoholic drinks with an ABV above 1.2%, the minimum threshold at which alcohol duty is payable. The information collected includes: the volume purchased in ml, product type, ABV and the brand. From this we can calculate the units of alcohol (1 UK unit is 8 mg or 10 mL of pure alcohol) contained in any alcoholic drinks purchased. We aggregate this data into 4‐week periods, across all households that participated in the panel for all 4 weeks of the period.

### 2.2 Defining Reformulation of a Product

We define a reformulation as a window of 6 consecutive 4‐weekly periods in which the ABV for a product is stable (no more than 0.01% variation in mean ABV across all purchases between periods), followed by a further 6 × 4‐week window in which the ABV is again stable, but at a new level that is at least 0.1 percentage points higher or lower than the previous stable ABV. In practice there may be a period of instability in the mean ABV between these windows, as retailers sell remaining stock of the original product and introduce the reformulated version. Therefore the two 6 x 4‐week windows with stable pre‐ and post‐reformulation ABVs do not need to be consecutive. Figure A7 presents an illustrated example of this definition.

This definition of a reformulation was included in the study protocol. We subsequently validated it against a set of known reformulations that we identified through analyses of the alcohol trade press [26]. This led to a minor change in the definition to avoid the exclusion of some known reformulations that took place within the last 6 months of 2025 and for which there was insufficient time to observe a 6 × 4‐week post‐reformulation period. The revised definition allowed a shorter post‐reformulation window of at least 3 consecutive 4‐week periods, where this includes the last period in the data.

The validation process identified two key considerations when using the Worldpanel data for the purposes of identifying reformulations. Firstly, products only appear in the dataset in a period if at least one participating household purchases them during that period. As a result, we may miss or delay identifying reformulations of some products with low sales volumes that appear in the data infrequently. Secondly, the level of detail available on each product in the data may mean that we are unable to separate some closely related product lines. This is particularly likely to be the case for wine, where the data includes the brand name and wine colour, but not grape type. For example, where a producer uses the same brand name for both a chardonnay and sauvignon blanc, we cannot identify these as separate products. This may mean that we are unable to identify reformulations that have happened for only one of a grouped set of products, or that we wrongly identify ABV fluctuations arising from shifts in the relative sales of the individual products as a reformulation. This is a more minor problem for other beverage types where products are typically clearly separated.

### 2.3 Analysis

Mean ABVs, both overall and by beverage type (beer, cider, wine, spirits and Ready‐To‐Drinks (RTD—pre‐mixed wine‐ or spirits‐based products)), were calculated for each period, weighted by the number of units in each purchase. Trends are assessed using the 12‐month trailing rolling average to remove the impact of seasonality. Due to the size of the dataset (6 million individual purchase records), upper and lower 95% confidence intervals are narrower than 0.03% and are therefore not presented (and, by implication, all changes of 0.1% or more are statistically significant).

For each identified reformulation, we calculate the market share of that product as the mean proportion of the total volume of pure alcohol purchased in the dataset within its beverage category across the 6 periods prior to the start of reformulation. We also record the proportions of product volume purchased and total value of purchases as secondary outcomes.

To explore the alignment between reformulations and the reforms to the alcohol duty system, we divide the data into 4 periods: (i) prior to October 2021, when the duty reforms were first published for consultation [9]; (ii) from October 2021 to March 2023 when the details of the reforms were confirmed [11]; (iii) between March 2023 and August 2023 when the reforms were implemented; and (iv) since August 2023. For the purpose of assigning reformulation to time periods, we consider reformulations to have occurred at the start of the first 4‐week period of the post‐reformulation window in which the ABV is stable at the new level.

There were two changes from the pre‐registered protocol: firstly, we included data from 2025, which was not available at the time of registration and secondly, we included an additional exploratory off‐protocol time series analysis to examine the significance of any changes in reformulation rates following the duty reforms (see Supporting Information for details).

### 2.4 Sensitivity Analyses

Given the data considerations outlined above, we test the robustness of our results to alternative definitions of a reformulation in a range of sensitivity analyses where we: (i) increase the tolerance for ABV variation within a period where the ABV is considered stable from 0.01% to 0.02%; (ii) reduce the required period with a stable ABV from 6 to 3 months; (iii) reduce the tolerance for ABV variation from 0.01% to 0.005%; and (iv) increase the required period with a stable ABV from 6 to 9 months.

## 3 Results

### 3.1 Changes in the Average ABV of Alcohol Products

**FIGURE 1:**
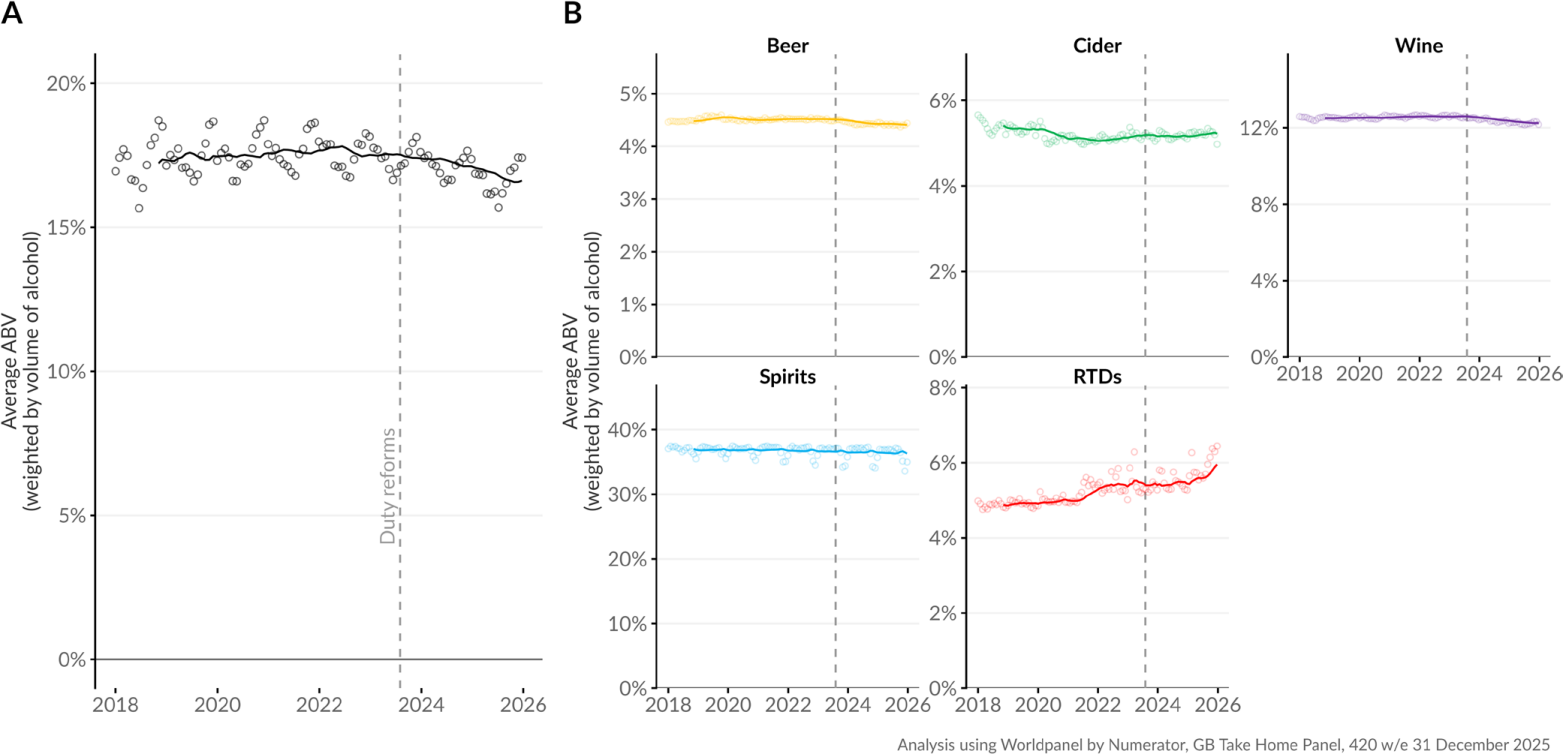
Mean alcohol‐by‐volume (ABV) of all alcoholic products (panel A) and by beverage type (panel B) by 4‐week period (hollow circles) and 12‐period trailing rolling average (solid line). Note the different *y*‐axis scales in panel B. RTD, Ready‐To‐Drink.

Overall the mean ABV of alcoholic products sold in the off‐trade in Great Britain has was broadly stable prior to the duty reforms, rising slightly from 17.2% in late 2018 to 17.7% in June 2022. Since the reforms, however average strengths have fallen, reaching 16.6% in late 2025. These trends are illustrated in Figure 1A, and by beverage type in Figure 1B, with annual averages shown in Table A1. The beverage‐specific trends show some heterogeneity, with notable changes in ABV occurring for all beverage types. The mean ABV of beer has fallen from 4.5% in 2022 to 4.4% in 2025, while the mean wine ABV has fallen from 12.6% to 12.3% over the same period. The mean ABV of spirits has also fallen from 36.5% in 2022 to 36.2% in 2025. Conversely, the mean ABV of cider has risen slightly from 5.1% to 5.2%, having previously fallen from 5.4% in 2018. The mean ABV of RTDs has been rising throughout the analysis period, from 4.9% in 2018 to 5.4% in 2022 and 5.9% in 2025.

### 3.2 Reformulations

There were 8811 unique products in the data. We identified 557 reformulations across 495 separate products (5.6% of all products) between 2018 and 2025, as summarised in Table 1. Over half of the reformulations identified were in wine products (50.4%), but cider had the greatest proportion of products that were reformulated at least once during the analysis period (9.7%).

**TABLE 1:** Summary of identified reformulations 2018–2025.

	Unique products	No. of reformulations	No. of unique products reformulated	Proportion of products reformulated at least once
Overall	8811	557	496	5.6%
Beer	2615	96	92	3.5%
Cider	661	67	64	9.7%
Wine	3053	281	235	7.7%
Spirits	1575	70	65	4.1%
RTDs	907	43	40	4.4%

Abbreviation: RTD, Ready‐To‐Drink.

Table 2 shows how reformulation patterns vary by type of alcohol and overall. This highlights that the number of reformulations per 4‐week period increased for all beverage types following the announcement of the reforms in October 2021, with the largest increase seen for beer, spirits and wine. The rate of reformulations then increased again, and to a greater extent, once the reforms were implemented in August 2023.

**TABLE 2:** Details of product reformulations by beverage type and time period. NAs represent periods with no reformulations for that beverage type.

	Beer	Cider	Wine	Spirits	RTDs
Reformulations per 4‐week period					
Before Oct 2021	0.3	0.3	1.7	0.3	0.1
Oct 2021—Feb 2023	0.7	0.4	2.6	0.7	0.2
Mar 2023—Jul 2023	1.5	0.0	2.0	0.8	0.0
Aug 2023 onwards	2.0	1.5	4.7	1.2	1.1
Overall 2018–2025	0.9	0.6	2.7	0.7	0.4
Average market share of reformulated products					
Before Oct 2021	0.58%	1.62%	0.03%	0.04%	0.29%
Oct 2021—Feb 2023	0.08%	0.71%	0.03%	0.05%	0.27%
Mar 2023—Jul 2023	0.05%	NA	0.04%	0.07%	NA
Aug 2023 onwards	0.49%	0.36%	0.04%	0.11%	0.46%
Overall 2018–2025	0.42%	0.64%	0.04%	0.08%	0.43%
Proportion of reformulations that reduced ABV					
Before Oct 2021	81.2%	92.3%	64.6%	93.8%	80.0%
Oct 2021—Feb 2023	83.3%	100.0%	58.7%	61.5%	75.0%
Mar 2023—Jul 2023	66.7%	NA	50.0%	66.7%	NA
Aug 2023 onwards	95.2%	91.5%	71.0%	73.7%	88.2%
Overall 2018–2025	89.6%	92.5%	66.5%	75.7%	86.0%
Average ABV change (percentage points)					
Before Oct 2021	−0.16%	−0.57%	−0.37%	−4.39%	−0.72%
Oct 2021—Feb 2023	−0.31%	−0.81%	−0.37%	−0.87%	−1.88%
Mar 2023—Jul 2023	0.00%	NA	−0.12%	−0.87%	NA
Aug 2023 onwards	−0.29%	−0.54%	−0.52%	−1.14%	−0.54%
Overall 2018–2025	−0.25%	−0.58%	−0.44%	−1.82%	−0.68%

Abbreviations: ABV, alcohol‐by‐volume; RTD, Ready‐To‐Drink.

There were fewer clear patterns in the market shares of reformulated products except that reformulated ciders, beers and RTDs generally represented a greater share of the market than the spirits and wines. The majority of reformulations were ABV reductions (76.3%), but ABV increases represented a larger proportion of changes for wine (33.5%) and spirits (24.3%) compared to RTDs (14.0%), beer (10.4%) and cider (7.5%). There is some evidence that reformulations after the duty reforms were more likely to be reductions compared to reformulations before the reforms for beers, wines and RTDs.

Finally, given typical product strengths, the 0.58 percentage point (pp) reduction in cider ABV in the average reformulation, and the 0.44 pp reduction for wine are particularly noteworthy, while the smaller 0.25 pp reduction for beer suggests more incremental shifts when beers are reformulated. There were no apparent shifts over time in the magnitude of ABV changes on reformulation.

**FIGURE 2:**
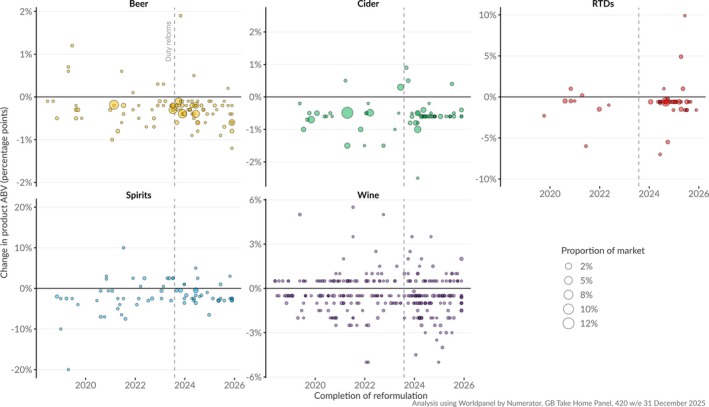
Timing, direction, magnitude and market share of all identified reformulations by beverage type. Note the different *y*‐axis scales between panels. ABV, alcohol‐by‐volume; RTD, Ready‐To‐Drink.

These patterns are illustrated visually in Figure 2, in which each reformulation is represented by a single bubble, sized by the market share of the reformulated product. This clearly highlights the clustering of ABV‐reducing reformulations for beer, cider and RTDs, and to a lesser extent spirits and wine, in the wake of the duty reforms in August 2023. Figure A8 shows a simple count of reformulations over time, while Figures A9 and A10 are alternative versions of Figure 2, showing the pre‐ and post‐reformulation ABVs for all identified changes. These illustrate clusters of reformulations since the duty reforms around 3.5% for beer, cider and RTDs and 11.5% for wine. The potential increase in reformulations following the duty reforms is corroborated by the exploratory statistical analysis, which found a significant increase (*p* < 0.001) of 6.1 additional reformulations per 4‐week period following the reforms (see Supporting Information for details).

### 3.3 The Size of the Below‐3.5% ABV Beer Market

**FIGURE 3:**
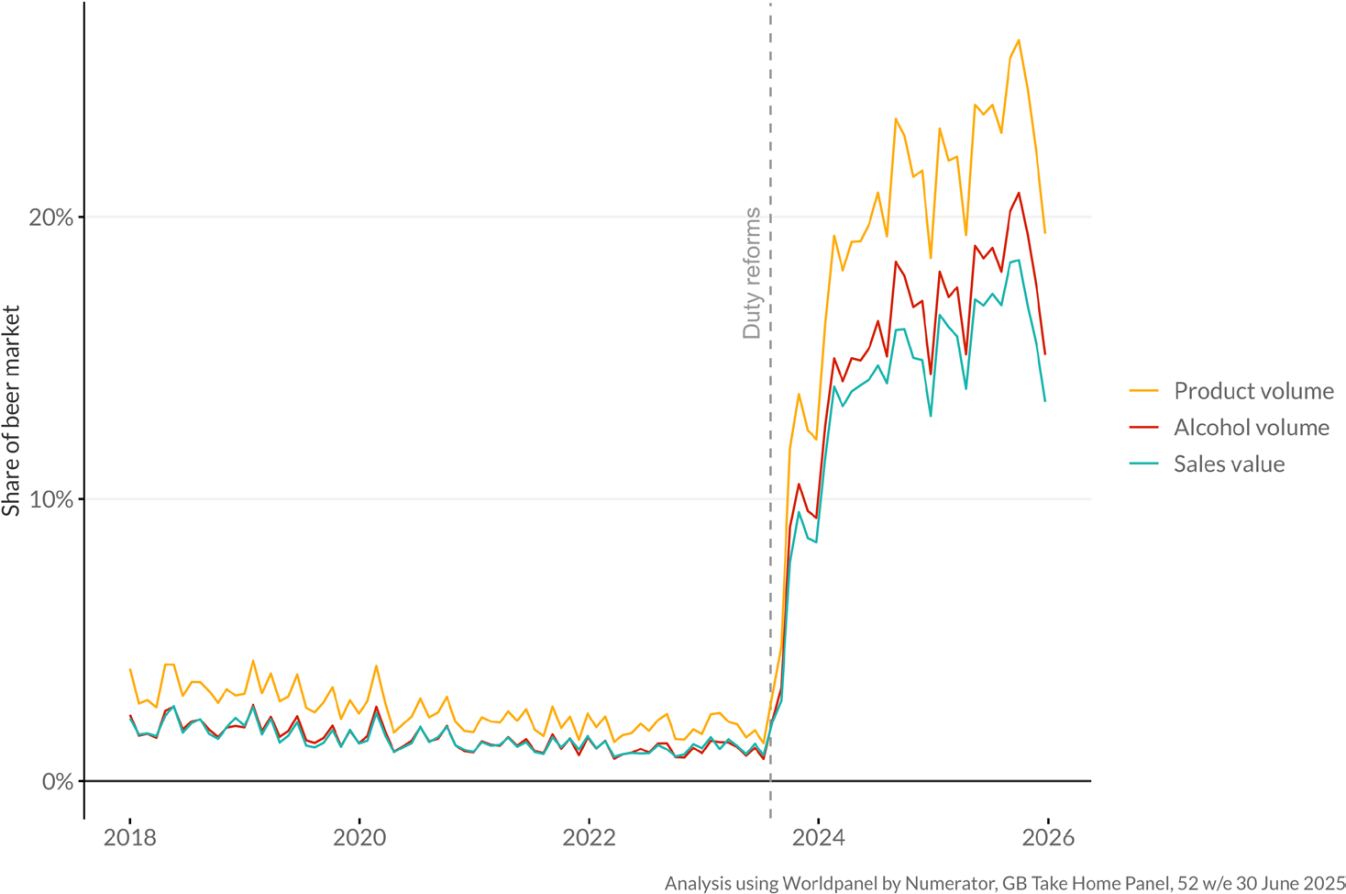
Proportion of beer in Great Britain being sold below 3.5% alcohol‐by‐volume.

The proportion of beer in Great Britain being sold between 1.2% and 3.5% ABV, by volume of alcohol, volume of product and sales value is shown in Figure 3. This shows a large increase in the size of this market across all measures following the increase in the lower‐strength beer duty threshold from 2.8% to 3.5% ABV in August 2023 as part of the duty reforms. Prior to this change, less than 3% of the beer market was sold below 3.5% ABV by any measure. By 2025, 18.1% of the alcohol sold in Great Britain as beer was below 3.5% ABV, equivalent to just under one‐quarter of the total volume of beer (23.0%) and around one‐sixth of the total expenditure on beer (16.4%).

Figure A1A breaks down this market growth from January 2023 onwards into new products launched after this date, sales growth for pre‐existing low ABV products and growth from products that have reformulated down into the < 3.5% ABV bracket, illustrating that the overwhelming majority of this growth has been driven by reformulation. Figures A12 and A13 show similar analyses for the 7.5%–8.5% and 8.5% + ABV beer market, which show no obvious signs of an increase in reformulation across this threshold.

### 3.4 Sensitivity Analyses

The impact of using alternative approaches to defining a reformulation is shown in Table A3 and Figures A15–A19. Adjusting the ABV tolerance for a product to be considered stable makes very little difference to our results, but changing the length of the period over which a product's ABV must be stable substantially changes the number of reformulations identified—reducing it from 557 to 315 for a longer period and increasing it to 815 for a shorter period. The vast majority of the difference is due to changes in the number of wine reformulations identified, suggesting that our estimate of the number of reformulated wine products is considerably more uncertain than for other beverage types. Under all sensitivity analyses the temporal pattern of the identified reformulations remains the same, with a notable increase in activity around the time of the duty reforms.

## 4 Discussion

Our analysis finds that there has been a more than 1 pp reduction in the average ABV of alcoholic products sold in the off‐trade in Great Britain between mid‐2022 and the end of 2025, driven by falls in the mean strengths of beer, wine and spirits. We also find widespread reformulation in the off‐trade alcohol market, with more than 1 in 20 products on sale changing their ABV at some point during the study period, and the majority of these reformulations leading to lower‐strength products. Although the announcement of the reforms in October 2021 preceded an increase in reformulations, the implementation of the reforms in August 2023 was followed by a substantial further increase in both the pace and scale of reformulations. The clearest impact of these reformulations is in a dramatic increase in the share of the beer market being sold at below 3.5% ABV from less than 3% to over 18%, although there have also been notable reformulations above this threshold. Reformulations are not solely restricted to beer, however, with wine seeing more than double the number of reformulations of any other beverage type, although limitations in the data mean these changes are much more uncertain.

There have been few previous studies on reformulation of alcoholic drinks. One study examined only reductions in ABV for beer in Great Britain between 2015 and 2018 using the same dataset and a similar definition of a reformulation, although excluding changes that increased the ABV. This identified 33 reformulations across the study period, equivalent to around 0.6 per 4‐week period [23]. This is higher than we find for 2018–2021, but still considerably lower than the 2.0 per 4‐week period we observe following the 2023 duty reforms. Another study used data from a supermarket website collected between May and October 2023 to explore the early impacts of the duty reforms, finding some evidence of reformulation, particularly for beer and spirits [22]. A wider body of literature has described the effect of policy decisions, such as the introduction of the Soft Drinks Industry Levy in the UK, on reformulation of non‐alcoholic drinks and food, finding that these policies can be effective at driving reformulation and improving public health [7, 8, 27].

This study used a large‐scale, nationally representative dataset of alcohol purchases to produce the first comprehensive analysis of recent alcohol reformulations in Great Britain. It also explored a potential cause of the reformulations by assessing their alignment with substantial reforms to the UK alcohol duty system. Although our analysis cannot demonstrate a causal link, the clear temporal association between the reforms and increased reformulation activity and the number of reformulations at ABV levels where new incentives to reformulate were introduced by the reforms strongly suggests that the reforms have played at least some role in driving an increase in reformulations. Our results are robust to a range of sensitivity analyses and we have also validated our approach to identifying reformulations against a list of known product reformulations reported in the retail trade press.

Our finding that duty reforms appear to have coincided with an increase in reformulations that reduce the ABV of alcohol products has important implications for public health. As an indication of the potential impacts, if we assume consumers did not otherwise change their purchasing behaviours (which is a strong and highly uncertain assumption), the 0.91 percentage point reduction in the mean ABV of alcohol sold in Great Britain between 2022 and 2025 would equate to 8.1 million fewer litres of alcohol being sold—around 15.1 units of alcohol per adult per year [28]. This change would be equivalent to a 2.7% reduction in mean weekly consumption [29]. This is in line with the stated aim of the duty reforms to reduce alcohol consumption by incentivising manufacture of lower‐strength alcoholic drinks [9, 30]. Previous research has highlighted the potential for reformulation to improve public health [31], while noting that achieving the large‐scale reformulation required to achieve meaningful health benefits may be challenging [32].

The sharp increase in purchases of beer at less than 3.5% ABV suggests a clear effect of the duty reforms, primarily through encouraging producers to lower the ABV of existing products to 3.4% to take advantage of the lower‐strength duty rate. This magnitude of market share of lower‐strength beer is unusual internationally, although it is notable that in both Sweden and Australia, below‐3.5% ABV beer makes up around a quarter of beer sales [33], suggesting that a large lower‐strength beer market is a sustainable goal. This finding is notable in the context of the European Union having changed their rules on alcohol taxation in 2020 to allow member states to offer lower rates of duty on beer at 3.5%, as opposed to 2.8% previously [34]. Our results suggest that tax incentives at this threshold can lead to substantial product reformulation, although current low rates of beer duty for many European countries may limit the effectiveness of this approach as it limits the scale of potential economic gains for producers [30].

As the data used in this analysis is taken from a sample of the general population, our coverage of purchases of lower sales volume products is somewhat limited, meaning that some reformulations may be identified as happening later than in reality, or missed entirely, as purchase records in the Worldpanel data may be too infrequent. However, because these products have low sales volumes, their impact on public health may be similarly limited. As discussed above, the way that wine products are identified in the data may also mean that we miss some reformulations and incorrectly classify shifts in sales as reformulations, although the fact that our core findings around the timing of reformulations remain unchanged in our sensitivity analyses even though the number of identified wine reformulations changes substantially provides confidence in our conclusions.

Another limitation is that we are only able to analyse changes in the ABV of products as reported to consumers on product packaging. UK food labelling regulations allow for margins of error around these stated ABVs of 0.3% for spirits, 0.5% for standard beer and wine, and 1% for strong beer and sparkling wine [35]. There is some limited evidence of producers both under‐ and over‐stating the true ABVs on product labels [36, 37]. Further research is needed to establish the extent to which true and stated ABVs vary when products reformulate.

Further research is also needed to understand the context of reformulation. This may include analyses using stronger causal inference methods to examine the role of the duty reforms in driving reformulation activity, how prices change when products reduce their ABV, and how consumers respond to the reformulation and its effect on sales. Understanding how reformulations map onto different groups of alcohol consumers is also critical to establish whether the changes since the duty reforms are likely to widen or narrow health inequalities. Finally, although we have observed a notable association between the duty reforms and rates of product reformulation, there are also other factors that may be influencing these changes, such as economic pressures on both producers and consumers as part of the ‘cost‐of‐living crisis’ as well as shifting consumer preferences towards lower‐strength products. It is also important to recognise that there is a history of alcohol and food industry actors using reformulation as a corporate tactic to distract from more effective policy solutions and position themselves as part of the solution, not part of the problem [5]. Therefore, assessing whether regulatory measures that incentivise reformulations lead to sustained reductions in the alcoholic strengths of products, even after the wider context changes, is important for understanding the benefits of such policies.

## 5 Conclusion

There have been substantial reformulations in the off‐trade alcohol market in Great Britain in recent years, with the pace of reformulations increasing substantially after the alcohol duty reforms in 2023. The reformulations have coincided with changes in the average strength of alcohol sold and rapid growth in the lower‐strength beer market. These results suggest that tax incentives can play an important role in encouraging the reformulation of alcohol products, although other contextual factors may also be important.

## Author Contributions

**Colin Angus:** conceptualisation, methodology, formal analysis, funding acquisition, visualisation, writing – original draft preparation. **Luke Wilson:** conceptualisation, methodology, writing – review and editing. **Sarah Jackson:** conceptualisation, methodology, writing – review and editing. **Nathan Critchlow:** conceptualisation, methodology, writing – review and editing. **Grace Leeming:** conceptualisation, methodology, writing – review and editing. **Jamie Brown:** conceptualisation, methodology, writing – review and editing. **John Holmes:** conceptualisation, methodology, funding acquisition, writing – review and editing.

## Funding

This work was supported by the National Institute for Health and Care Research (grant NIHR156679). Purchase of the Worldpanel by Numerator data was funded by the National Institute for Health and Care Research (grant NIHR135310). The views expressed are those of the authors and not necessarily those of the NIHR or the Department of Health and Social Care.

## Disclosure

Each author certifies that their contribution to this work meets the standards of the International Committee of Medical Journal Editors.

## Conflicts of Interest

The authors declare no conflicts of interest.

## Supporting information


**Figure A1.** Duty payable per unit of alcohol by product type under the old (panel A) and reformed (panel B) alcohol duty systems.
**Figure A2.** Alcohol duty payable on a 440 mL can of beer under the old and new alcohol duty systems (top panel) and the pre‐reform distribution of beer sales by alcohol‐by‐volume (ABV) (bottom panel).
**Figure A3.** Alcohol duty payable on a 440 mL can of cider under the old and new alcohol duty systems (top panel) and the pre‐reform distribution of cider sales by alcohol‐by‐volume (ABV) (bottom panel).
**Figure A4.** Alcohol duty payable on a 750 mL bottle of wine under the old and new alcohol duty systems (top panel) and the pre‐reform distribution of wine sales by alcohol‐by‐volume (ABV) (bottom panel).
**Figure A5.** Alcohol duty payable on a 700 mL bottle of spirits under the old and new alcohol duty systems (top panel) and the pre‐reform distribution of spirits sales by alcohol‐by‐volume (ABV) (bottom panel).
**Figure A6.** Alcohol duty payable on a 275 mL bottle of Ready‐To‐Drink (RTD) under the old and new alcohol duty systems (top panel) and the pre‐reform distribution of RTD sales by alcohol‐by‐volume (ABV) (bottom panel).
**Figure A7.** Examples of product‐level mean alcohol‐by‐volume (ABV) trajectories, with reformulation periods (highlighted in purple) between windows of ABV stability.
**Figure A8.** Timing and number of identified reformulations by beverage type.
**Figure A9.** Timing, direction, magnitude and market share of all identified reformulations by beverage type. Each bubble represents a reformulation, and the post‐reformulation alcohol‐by‐volume (ABV), while the other end of the line attached to each bubble represents the pre‐reformulation ABV—i.e. a longer line reflects a greater ABV change.
**Figure A10.** Timing, direction, magnitude and market share of all identified wine reformulations. Grey shaded area highlights the 11.5%–14.5% alcohol‐by‐volume (ABV) band affected by the ‘wine easement’.
**Figure A11.** Lower‐strength (< 3.5%) beer market broken down by product history.
**Figure A12.** Share of beer market (by alcohol volume) accounted for by 7.5%–8.5% and 8.5% + alcohol‐by‐volume (ABV) products.
**Figure A13.** Distribution of the beer market (by alcohol volume sold) across alcoholic strength bands.
**Figure A14.** Modelled (blue) and observed (red) counts of product reformulations per 4‐week period.
**Figure A15.** Density plot showing the distribution over time of reformulations under alternative approaches to defining a reformulation.
**Figure A16.** Timing, magnitude and market share of identified reformulations by beverage type, using a wider tolerance for alcohol‐by‐volume (ABV) stability.
**Figure A17.** Timing, magnitude and market share of identified reformulations by beverage type, using a shorter period for alcohol‐by‐volume (ABV) stability.
**Figure A18.** Timing, magnitude and market share of identified reformulations by beverage type, using a wider tolerance for alcohol‐by‐volume (ABV) stability.
**Figure A19.** Timing, magnitude and market share of identified reformulations by beverage type, using a longer period for alcohol‐by‐volume (ABV) stability.
**Table A1.** Average alcohol‐by‐volume (ABV) (weighted by alcohol volume) by beverage category and year.
**Table A2.** SARIMA model coefficients, standard errors and *p*‐values.
**Table A3.** Number of reformulations identified under alternative approaches to defining a reformulation.

## Data Availability

Use of the Worldpanel data is allowed under the terms of the contract and nondisclosure agreement between Numerator and the University of Sheffield, which requires research outputs to be submitted to the data provider ahead of publication. The data providers' right to request changes is limited to matters of accuracy regarding the data. The data provider played no further role in the research process, including in conception, design, analysis, interpretation, write‐up or the decision to publish. All analysis and interpretation were conducted independently of Worldpanel by Numerator. Worldpanel cannot independently verify the findings, nor can it endorse the views or findings of this report. Data used in these analyses are a commercial product licensed for use by the University of Sheffield and cannot be shared. The data are available for in‐person inspection at the University of Sheffield by researchers on reasonable request. Analytical code can be shared on request.
