# Supplementary material for: How Have Alcohol Producers Changed the Alcohol Content of Their Products? A Descriptive Analysis of Reformulation in the Off‐Trade Alcohol Market in Great Britain, 2018–2025
Source: Drug Alcohol Rev. 2026 Jun 30;45(5):e70197. doi: 10.1111/dar.70197 (PMC13316727; doi:10.1111/dar.70197)

**How have alcohol producers changed the alcohol content of their products? A descriptive analysis of reformulation in the off-trade alcohol market in Great Britain, 2018-2024 – Supplementary material**

Figure A1 - Duty payable per unit of alcohol by product type under the old (panel A) and reformed (panel B) alcohol duty systems


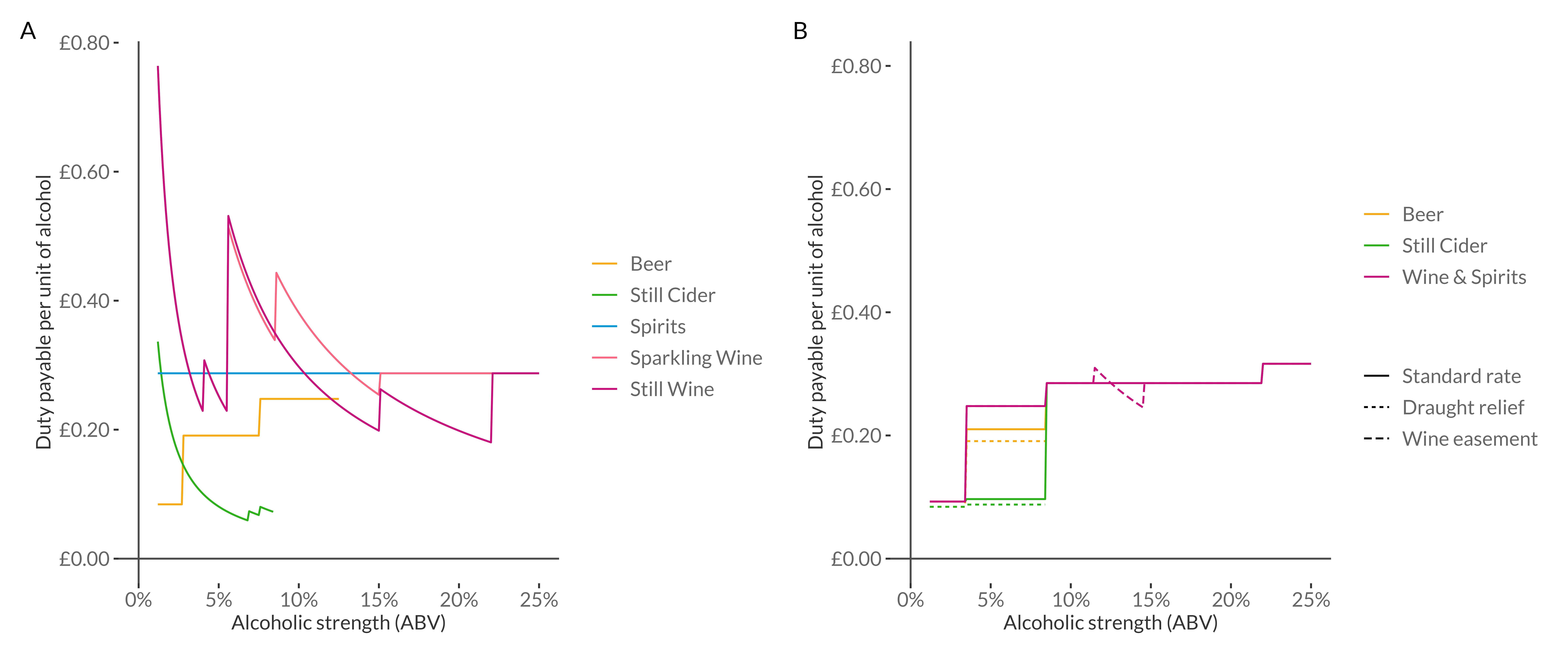


Figures A2-6 show, in the top panel, the total duty payable for 5 typical/illustrative products, selected as the modal serving size for each drink category in the Worldpanel data, and how this varies with the alcohol-by-volume (ABV) of the product, under the old and new alcohol duty systems. This illustrates both the differences between the two systems, and the incentives for reformulation under each system – i.e. the points in the ABV scale where a small change in ABV can lead to a change in the duty payable.

The bottom panel of each Figure shows the density of sales of that beverage type by ABV in the 12 months prior to the duty reforms (i.e. August 2022-July 2023) – i.e. what proportion of the market was being sold pre-reform at different ABV levels. These combined plots allow a comparison of the ABV thresholds at which the greatest incentives for reformulation exist, with the proportion of the market that is being sold at close to those thresholds. Using them it is possible to highlight several points where the combination of a substantial reformulation incentive and a significant proportion of the pre-reform market being sold at close to the relevant ABV threshold suggest that there is potential for reformulation to occur.

Figure A2 shows these plots for beer and illustrates several important features. Firstly, the duty payable on a typical 440ml can of beer is broadly similar under the old and new systems, albeit slightly higher at most ABV levels under the new system. In general both lines slope upwards, indicating that higher ABVs are associated with higher duty burdens. This means that there was previously, and remains under the new system, a small incentive for producers to reduce the ABV of their products, as this reduces the duty payable on each can.

The biggest differences between the old and new duty systems lies in the 2.8-3.5% and 7.5-8.5% ABV ranges, as a result of the increases in the thresholds for the lower- and higher-strength duty rates. This means that where previously there was a larger incentive to reduce the ABV of products from 2.8% to 2.7%, under the new system this is shifted to a reduction from 3.5% to 3.4%. The total duty payable under the new duty system on a 440ml can of 3.5% beer is 33.5p, while at 3.4% it is 14.4p, a saving to producers of 19.2p. There is a similar incentive for reformulation under the new system from 8.5% (duty payable of £1.11 per can) to 8.4% (duty payable of £0.81 per can). However, the bottom density plot illustrates that a substantial proportion of the beer market was being sold above the 3.5% threshold, while very little was being sold above 8.5%. This suggests that we might expect to see reformulation across the 3.5% threshold to a much greater degree than the 8.5% threshold.

Figure A2 - Alcohol duty payable on a 440ml can of beer under the old and new alcohol duty systems (top panel) and the pre-reform distribution of beer sales by alcohol-by-volume (ABV) (bottom panel)


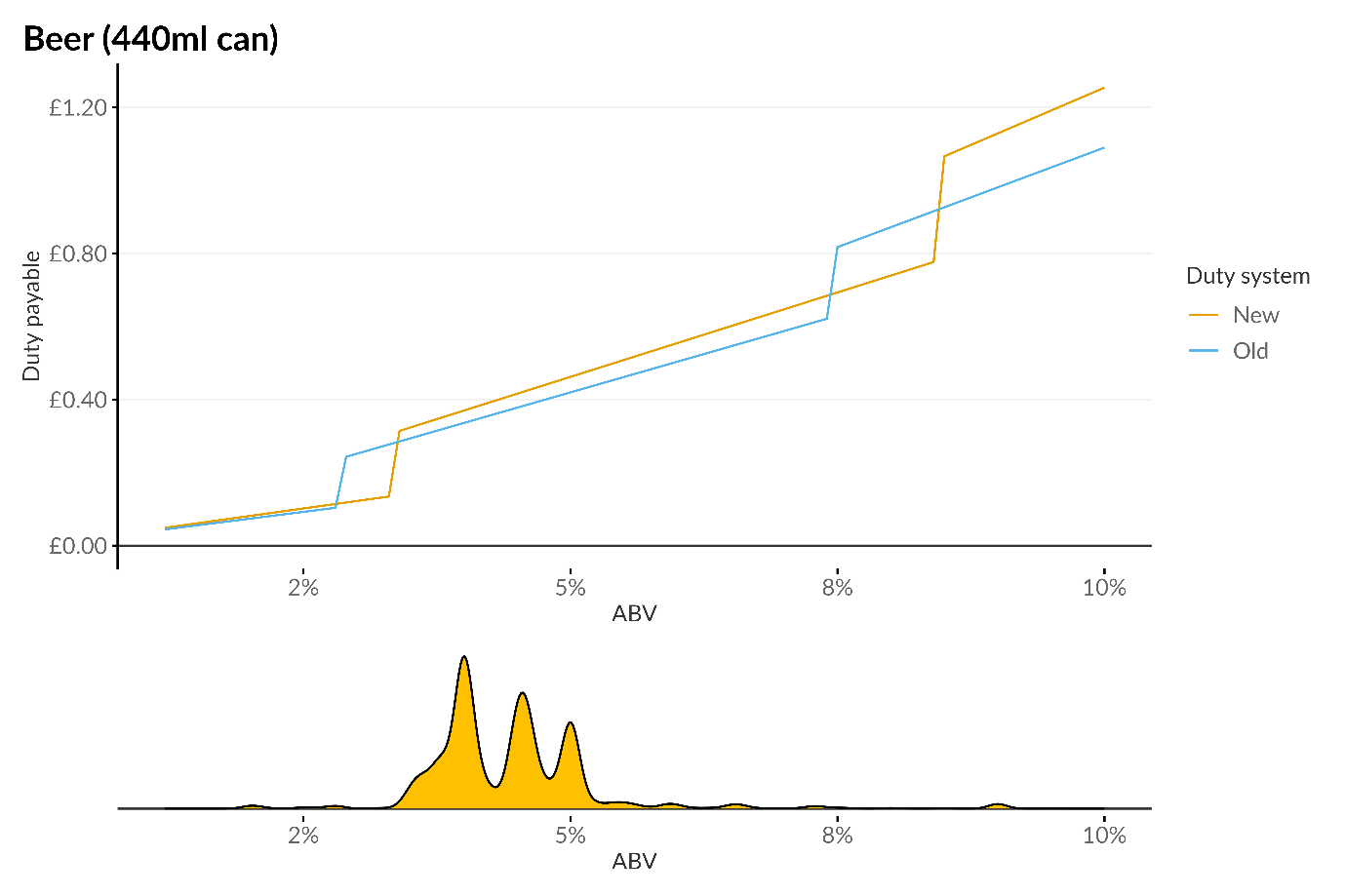


For cider, shown in Figure A3, the picture is quite different. Under the old system, there was no incentive for reformulation below 6.9% ABV – the duty payable on a 440ml can of 3.5% cider was precisely the same as on a 6.5% can. The reformed duty system introduced a slope – an incentive for reformulation across the ABV spectrum as stronger products now attract more duty per can. There is also a small threshold at 3.5%, as for beer, but the marginal incentive for reformulation to 3.4% is much less – a reduction in duty payable from 15.4p per 440ml can to 14.4p. However, as for beer, a substantial proportion of the cider market was sold relatively near to this ABV threshold. It is also notably that the overall duty payable on stronger ciders saw a large relative increase with the reforms, so it is possible that some producers may respond to this by reformulating to a lower ABV to mitigate the impact of the duty increase.

Figure A3 - Alcohol duty payable on a 440ml can of cider under the old and new alcohol duty systems (top panel) and the pre-reform distribution of cider sales by alcohol-by-volume (ABV) (bottom panel)


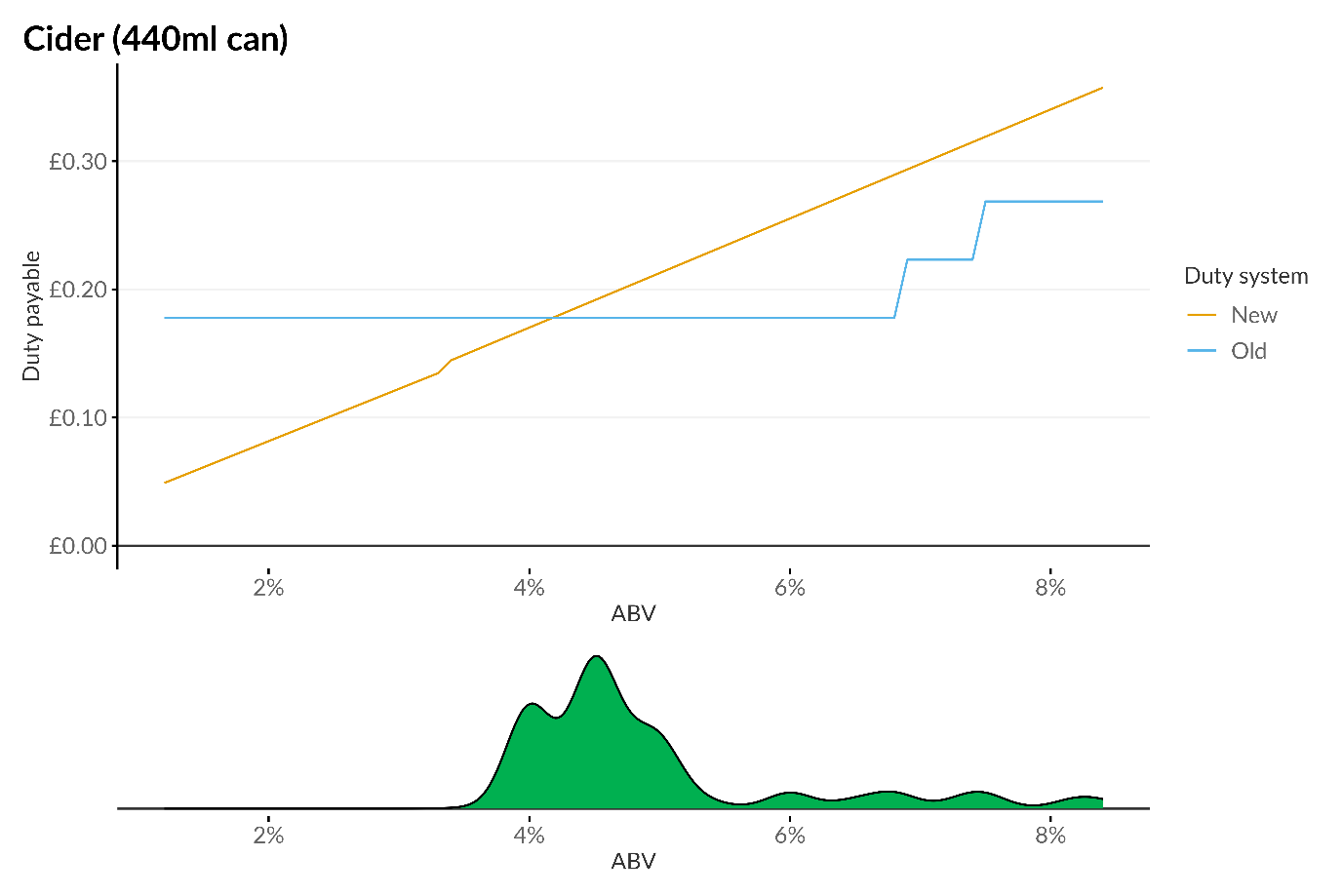


Wine, as illustrated in Figure A4, shows a different pattern again. Under the old duty system there was no incentive for reformulation between 5.5% and 15%, with all wine within this range attracting the same duty per bottle. The revised system introduces as slope across most of the ABV spectrum, giving producers an incentive to lower ABVs, however the ‘wine easement’ that was in place until February 2025 applied a flat duty level to a bottle of wine sold between 11.5% and 14.5%, removing (at least temporarily) any incentive for reformulation within this range. However, this did lead to clear thresholds at 11.5% and 14.5%, where a small ABV reduction could give duty savings. For example, a 700ml bottle of wine at 11.5% attracted duty of £2.77, compared to £2.53 for a bottle at 11.4%, a saving to producers of 24p. The density plot illustrates that these was little wine being sold pre-reform just above the 14.5% threshold (or the other thresholds at 3.5% and 8.5%), but a substantial proportion of the market was being sold close to the 11.5% threshold, suggesting potential for reformulation there.

Figure A4 - Alcohol duty payable on a 750ml bottle of wine under the old and new alcohol duty systems (top panel) and the pre-reform distribution of wine sales by alcohol-by-volume (ABV) (bottom panel)


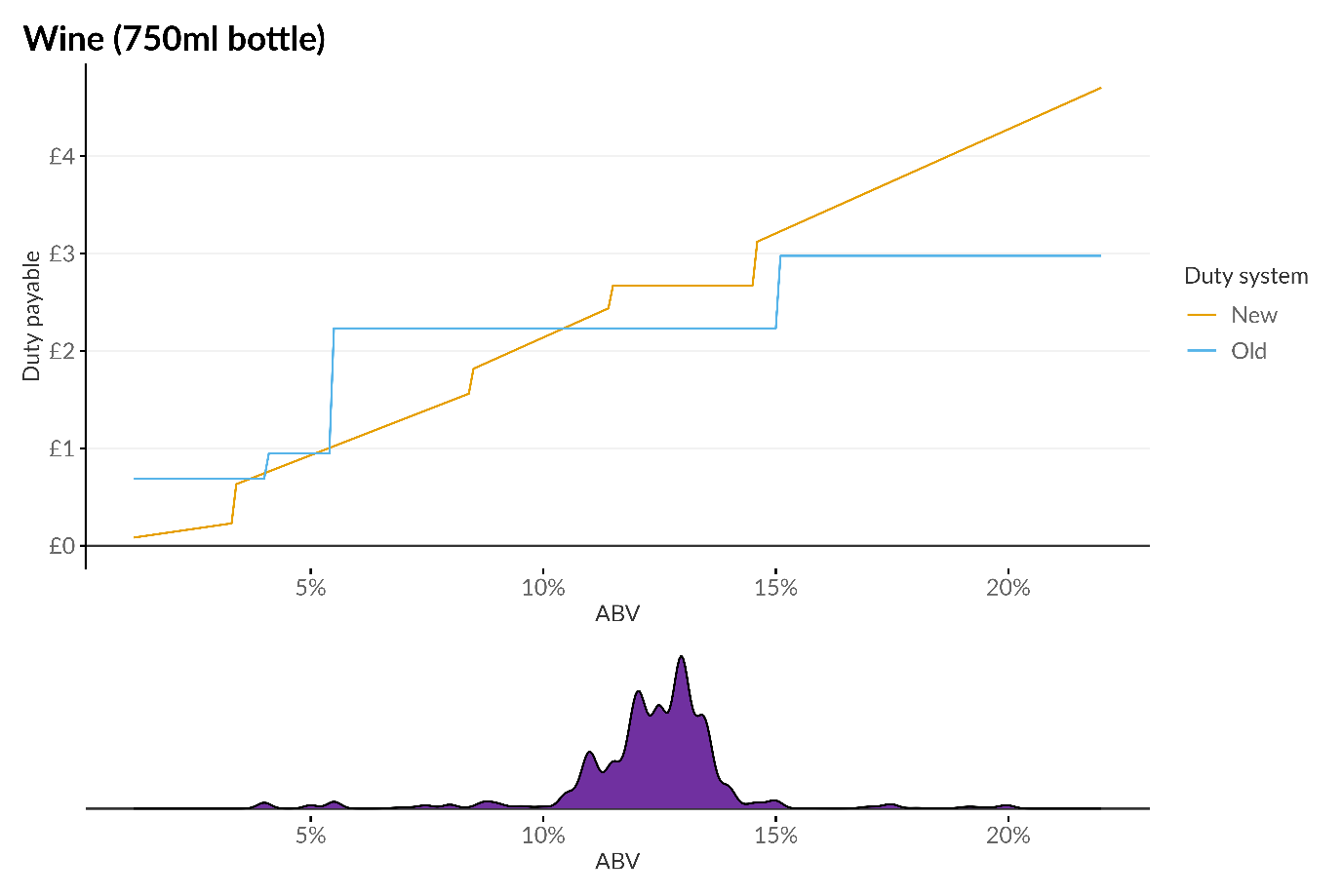


Spirits, as shown in Figure A5, present a simpler picture. Both the old and new system display a slope, with higher duty payable on a bottle of stronger spirits, but the only threshold introduced in the new system, at 22%, is at a point in the ABV distribution that the density plot shows almost no spirits were being sold close to.

Figure A5 - Alcohol duty payable on a 700ml bottle of spirits under the old and new alcohol duty systems (top panel) and the pre-reform distribution of spirits sales by alcohol-by-volume (ABV) (bottom panel)


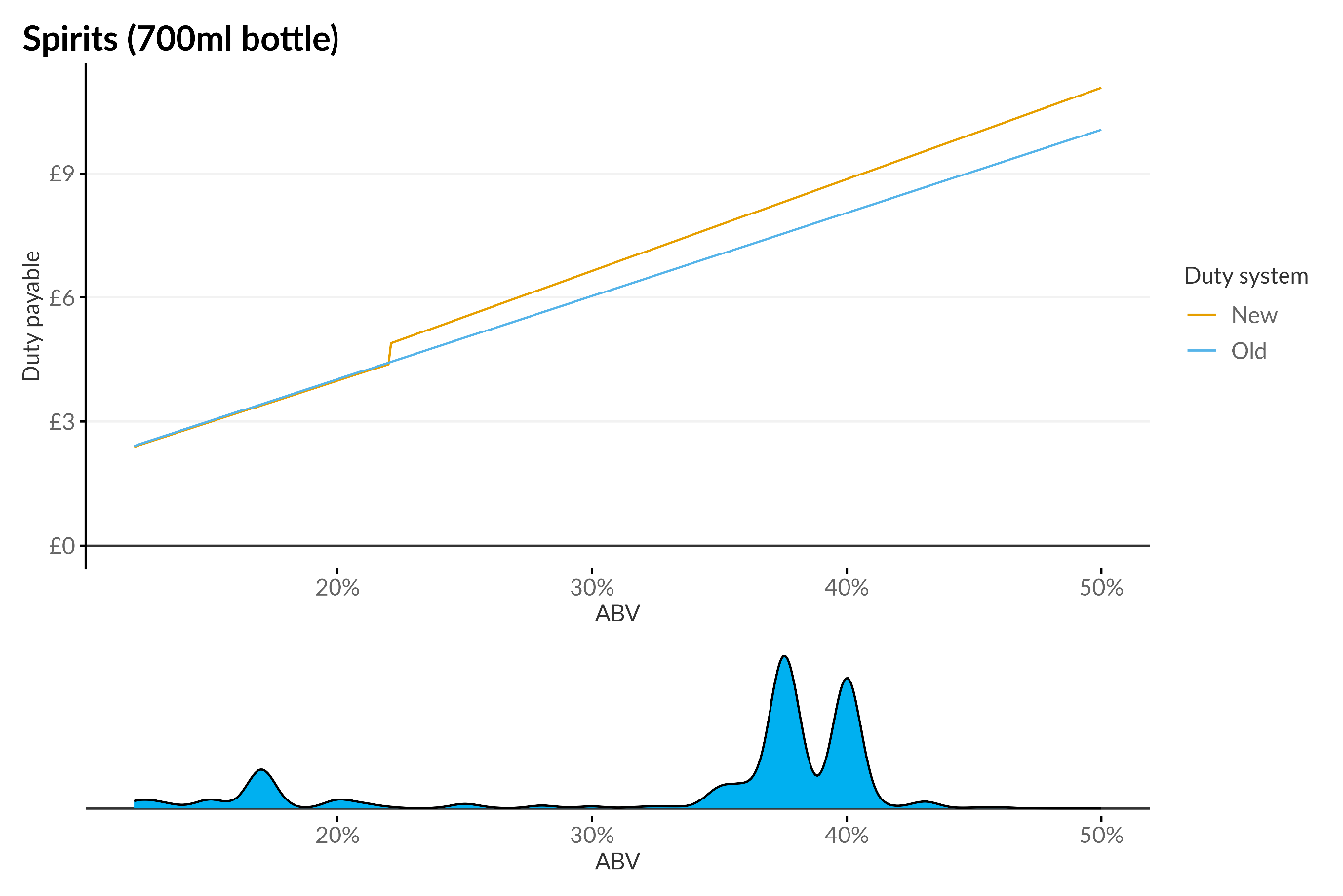


Finally, Figure A6 shows the similar plot for RTDs. This demonstrates a clear, and large, threshold at 3.5% under the new system that was not present previously. Under the new duty system, a typical 275ml bottle of RTD sold at 3.5% ABV would attract 24.7p in duty, compared to 9.0p for a bottle at 3.4%, a saving to producers of 15.7p. The density plot illustrates that pre-reforms the majority of RTDs were sold at close to 4% or 5%, meaning that there is potential for reformulation for a significant segment of the market.

Figure A6 - Alcohol duty payable on a 275ml bottle of RTD under the old and new alcohol duty systems (top panel) and the pre-reform distribution of Ready-To-Drink (RTD) sales by alcohol-by-volume (ABV) (bottom panel)


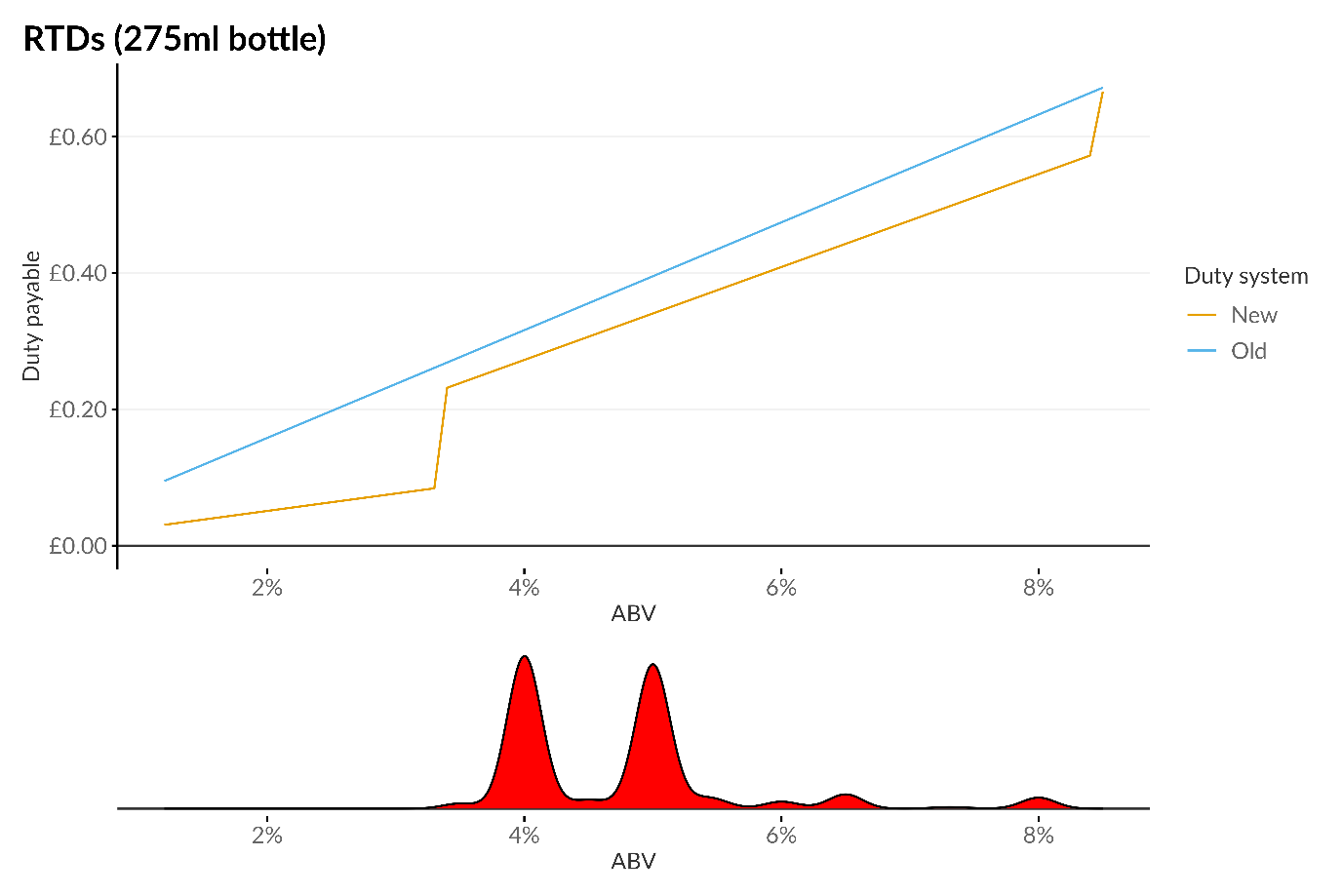


Figure A7 - Examples of product-level mean alcohol-by-volume (ABV) trajectories, with reformulation periods (highlighted in purple) between windows of ABV stability


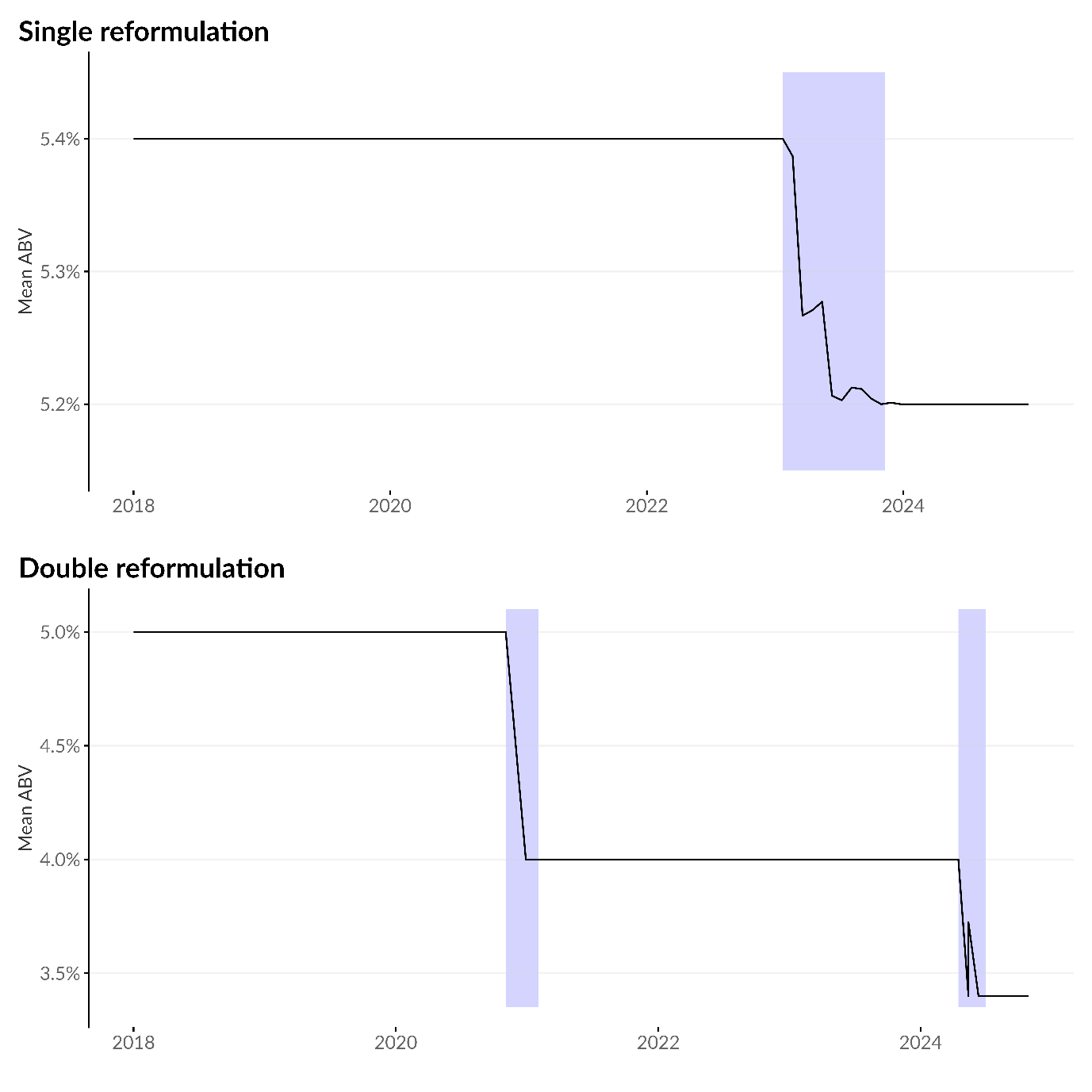


Table A1 – Average alcohol-by-volume (ABV) (weighted by alcohol volume) by beverage category and year


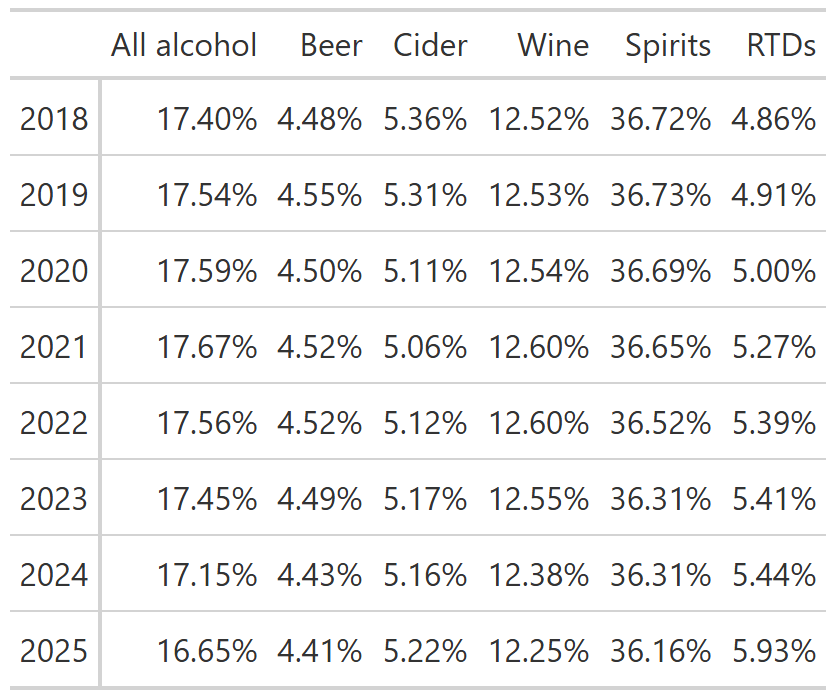


Figure A8: Timing and number of identified reformulations by beverage type


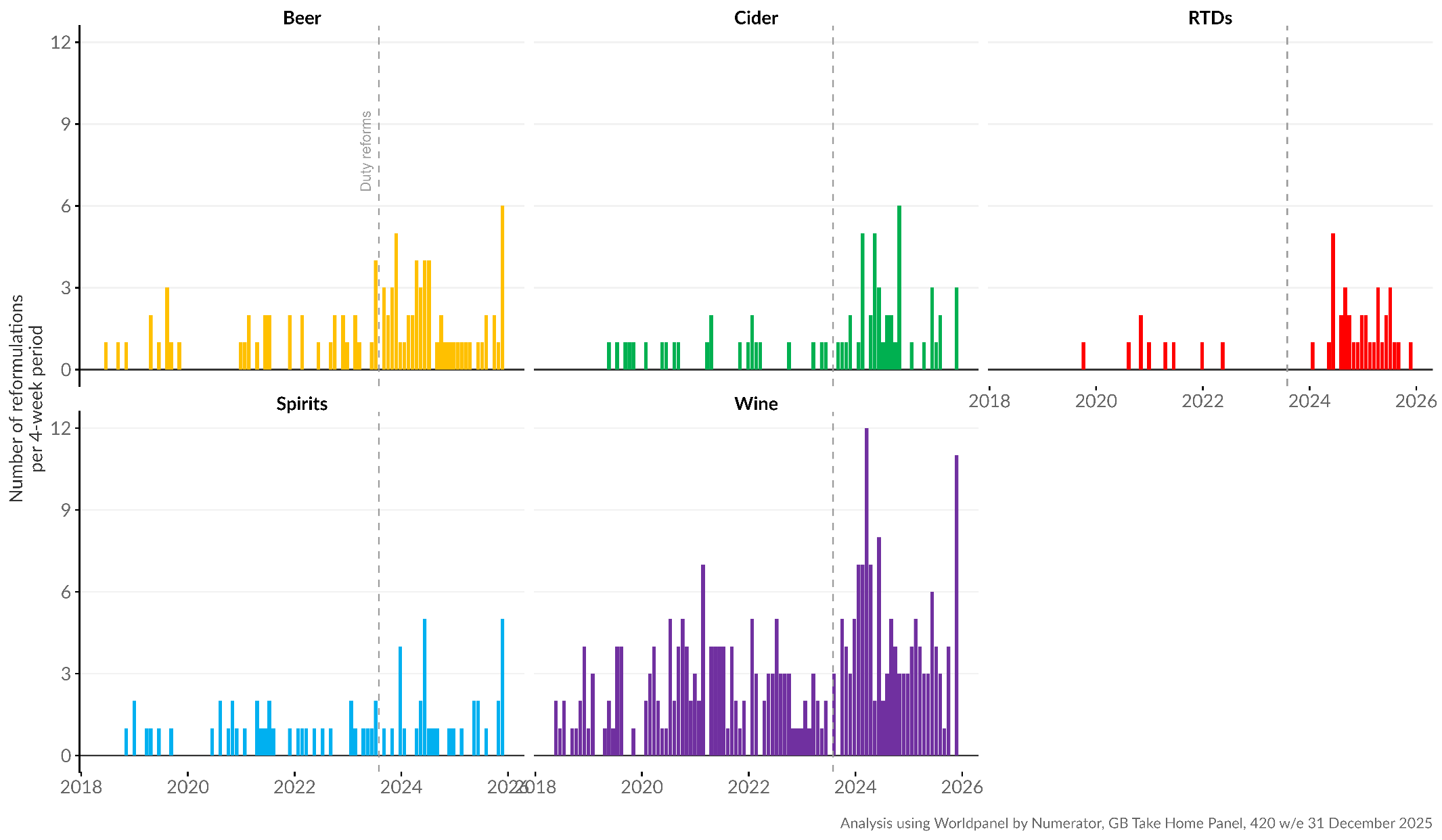


Figure A9 - Timing, direction, magnitude and market share of all identified reformulations by beverage type. Each bubble represents a reformulation, and the post-reformulation alcohol-by-volume (ABV), while the other end of the line attached to each bubble represents the pre-reformulation ABV – i.e. a longer line reflects a greater ABV change


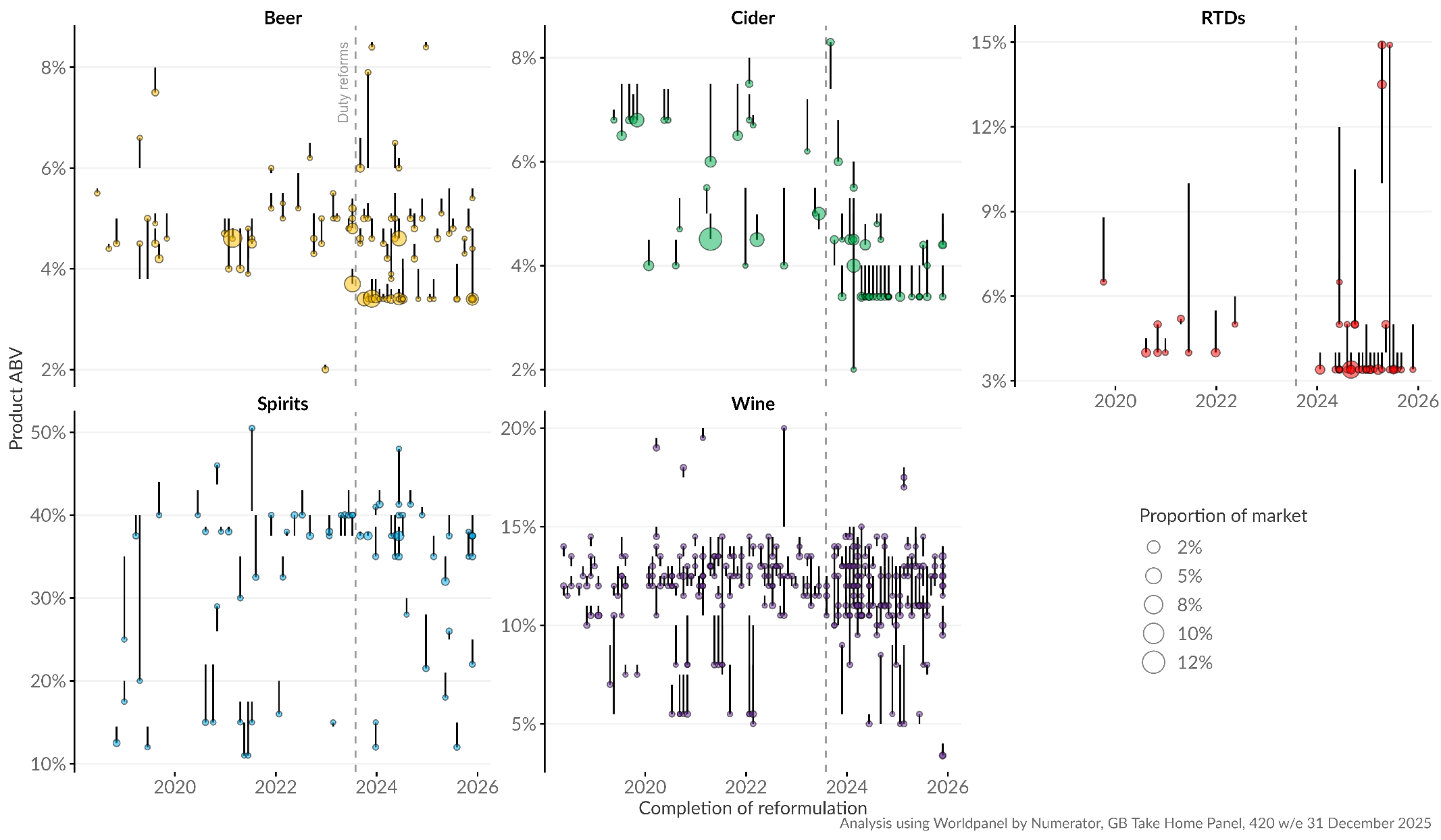


Figure A10 - Timing, direction, magnitude and market share of all identified wine reformulations. Grey shaded area highlights the 11.5-14.5% alcohol-by-volume (ABV) band affected by the ‘wine easement’


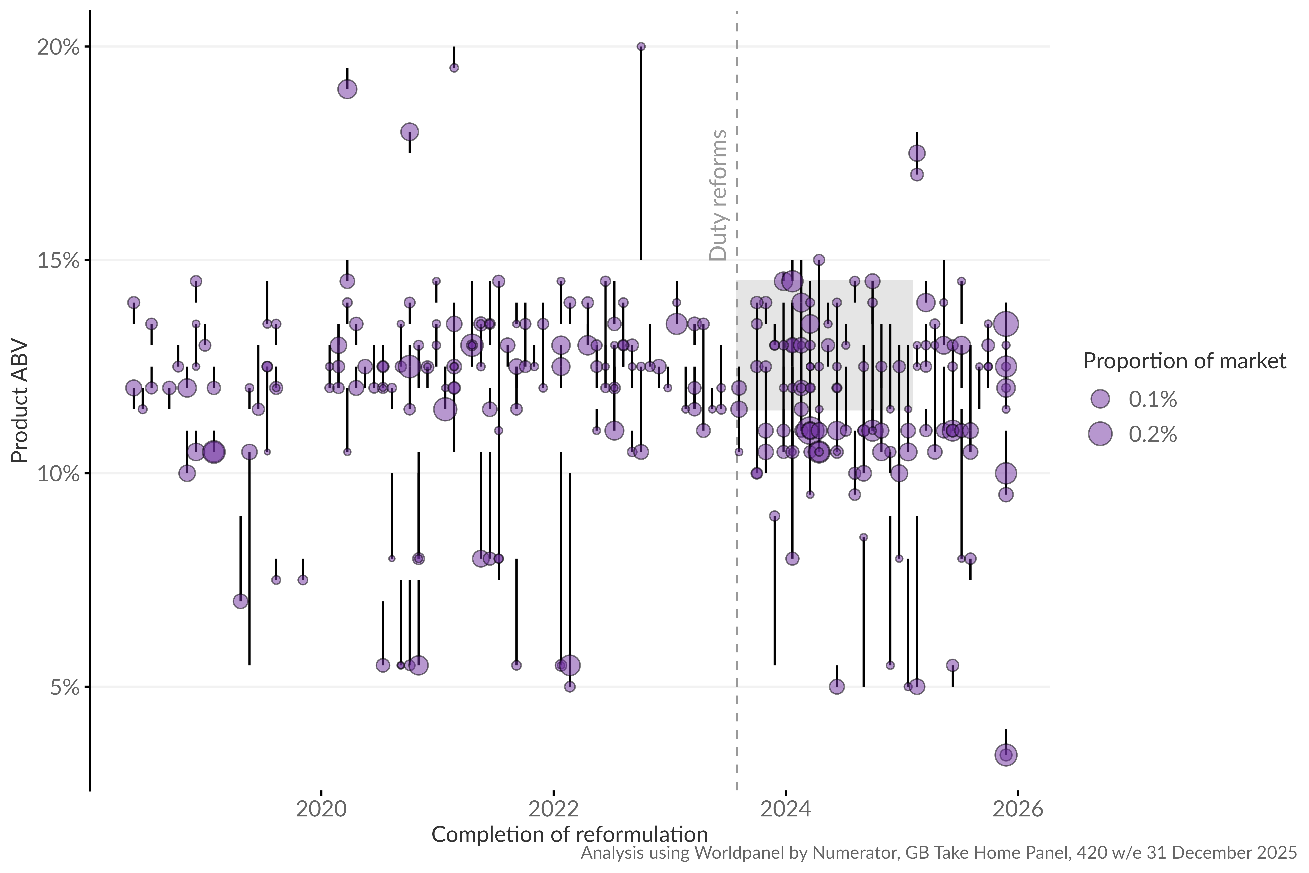


Figure A11 - Lower-strength (<3.5%) beer market broken down by product history


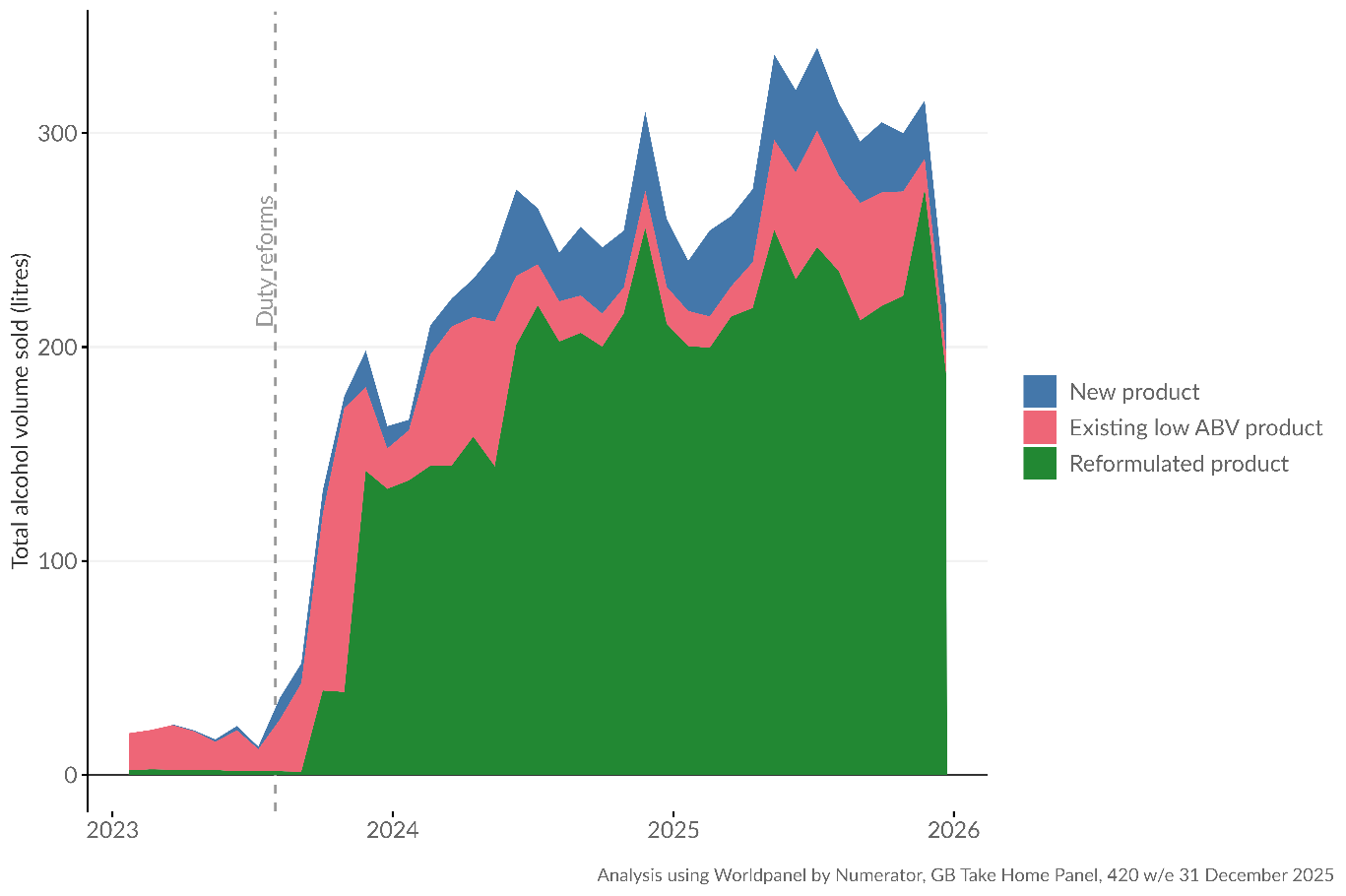


Figure A12 - Share of beer market (by alcohol volume) accounted for by 7.5-8.5% and 8.5%+ alcohol-by-volume (ABV) products


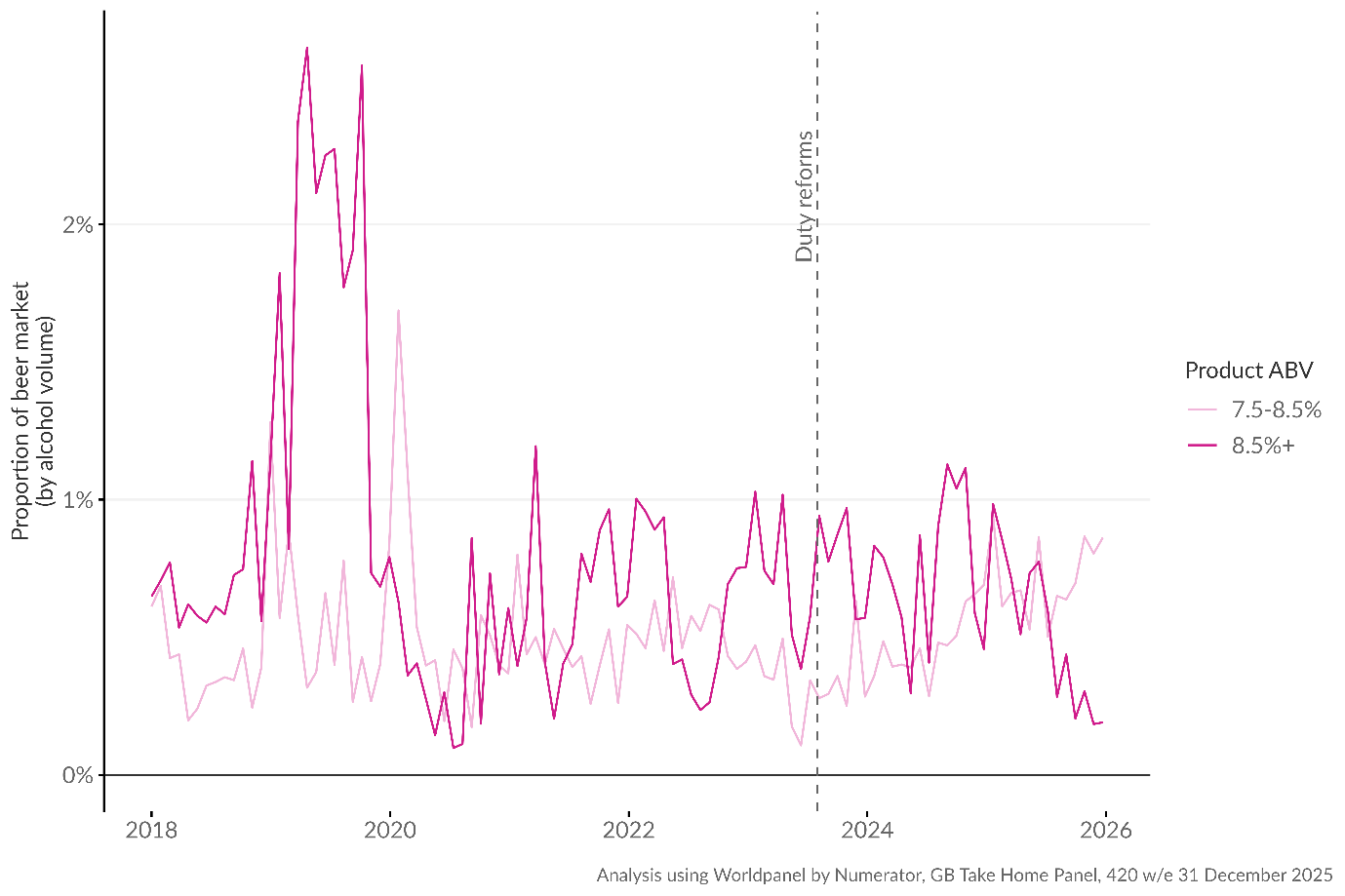


Figure A13 - Distribution of the beer market (by alcohol volume sold) across alcoholic strength bands


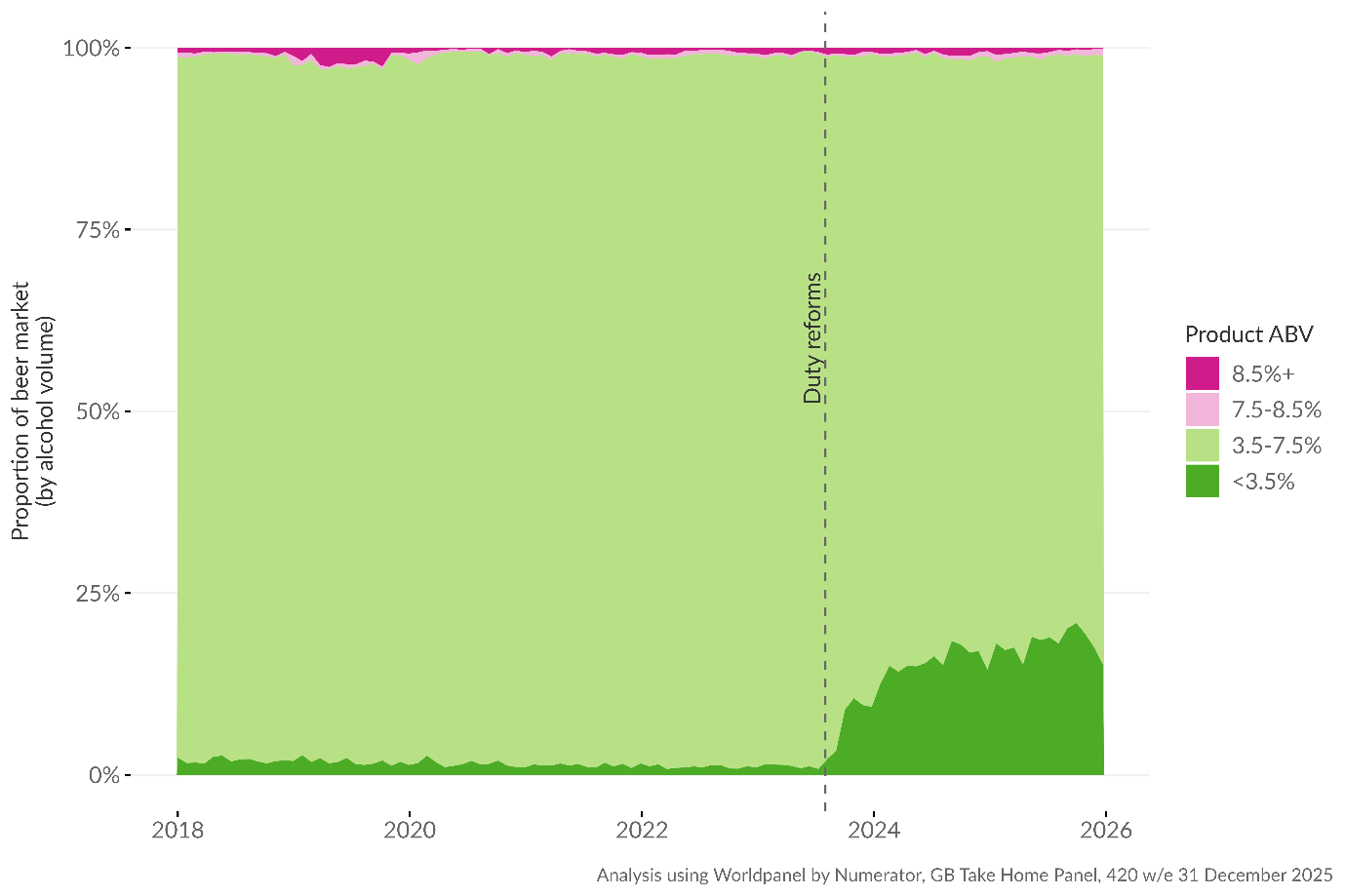


**Exploratory statistical modelling**

In an unplanned additional analysis, we used a Seasonal Autoregressive Integrated Moving Average (SARIMA) model to assess the number of reformulations per 4-week period and examine whether there was a significant change in this number following the August 2023 duty reforms. To do this we fitted models on the number of reformulations, with an additional dummy variable taking a value of 0 prior to the reforms and 1 thereafter. The coefficient on this dummy can therefore be interpreted as the number of additional reformulations per 4-week period following the implementation of the reforms.

Model specification was identified through examination of autocorrelation and partial autocorrelation plots as well as inspection of Bayesian Information Criterion (BIC) for comparison of alternative models. The final model specification was an ARIMA(1,0,1) SARIMA(1,0,0,12) model with a period of 13 to align with the 4-weekly data window.

The model, detailed below, suggests that the reforms were associated with a statistically significant increase of 6.1 additional reformulations per 4-week period.

We did not fit beverage-specific models due to the large number of zero counts for some beverage types.

Model coefficients:

Table A2: SARIMA model coefficients, standard errors and p-values

| Coefficient | Value | Standard error | p-value |
| --- | --- | --- | --- |
| AR1 | 0.7255 | 0.2516 | 0.0039 |
| MA1 | -0.4822 | 0.3183 | 0.1237 |
| SMA1 | 0.1816 | 0.1071 | 0.0901 |
| Intercept | 3.5030 | 0.7015 | 0.0000 |
| Reform dummy | 6.1131 | 1.1813 | 0.0000 |

Figure A14: Modelled (blue) and observed (red) counts of product reformulations per 4-week period


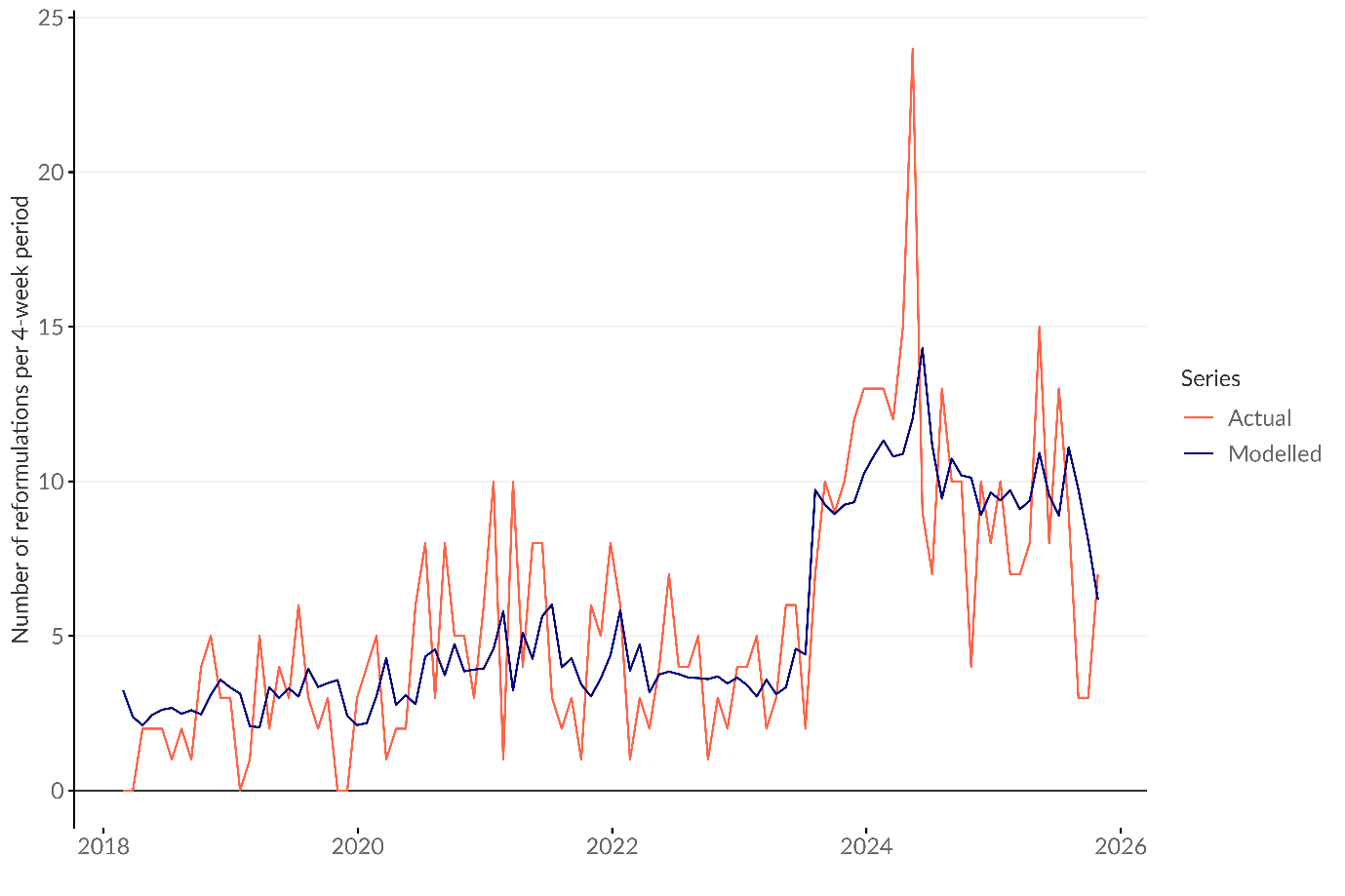


**Sensitivity analyses**

Table A3 – Number of reformulations identified under alternative approaches to defining a reformulation


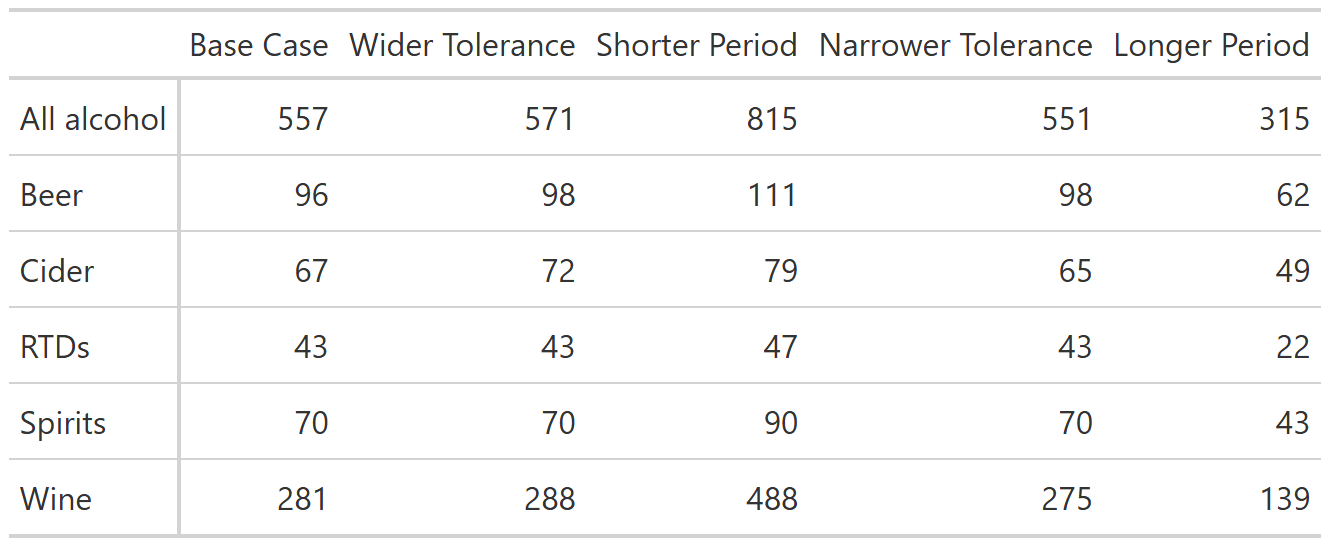


Base case – our primary analysis, Wider Tolerance – the ABV variation allowed within a period of stability before or after a reform is relaxed from 0.01pp to 0.02pp, Shorter Period – the duration of a period of stability is reduced from 6 to 3 months, Narrower Tolerance – the ABV variation allowed within a period of stability is tightened to 0.005pp, Longer Period – the duration of a period of stability required before and after a reformulation is increased to 9 months.

Figure A15 – Density plot showing the distribution over time of reformulations under alternative approaches to defining a reformulation


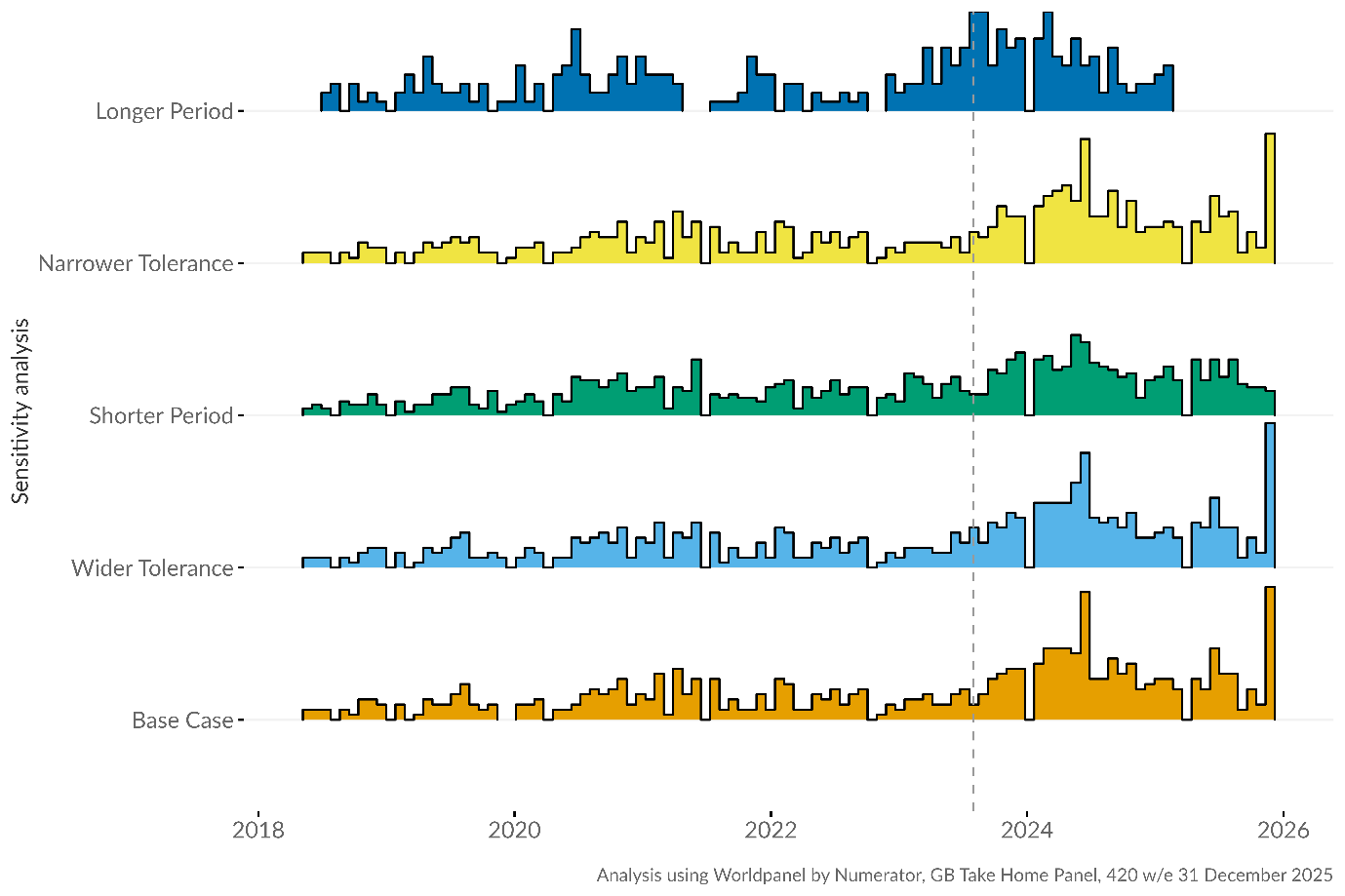


Figure A16 - Timing, magnitude and market share of identified reformulations by beverage type, using a wider tolerance for alcohol-by-volume (ABV) stability


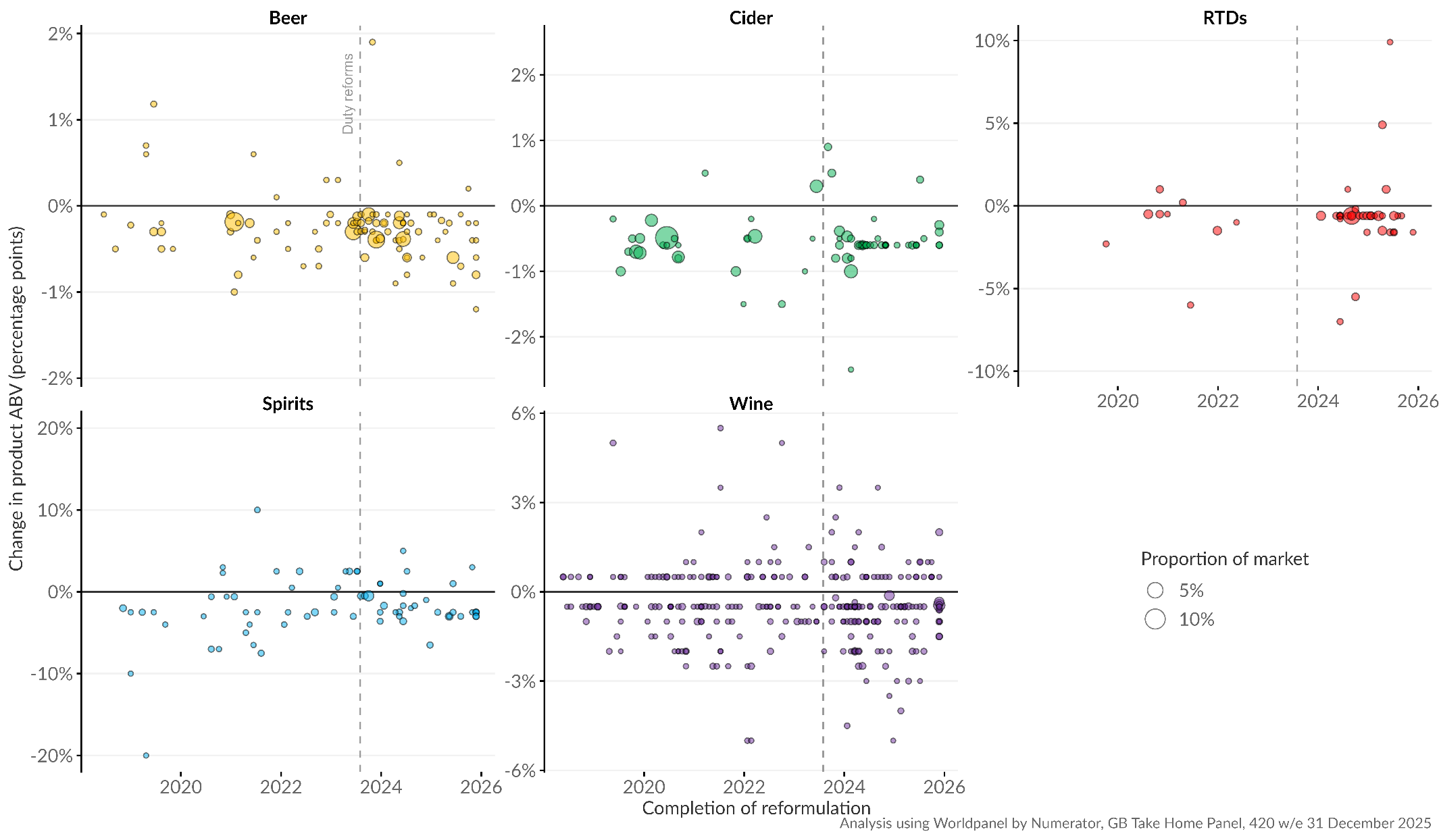


Figure A17 - Timing, magnitude and market share of identified reformulations by beverage type, using a shorter period for alcohol-by-volume (ABV) stability


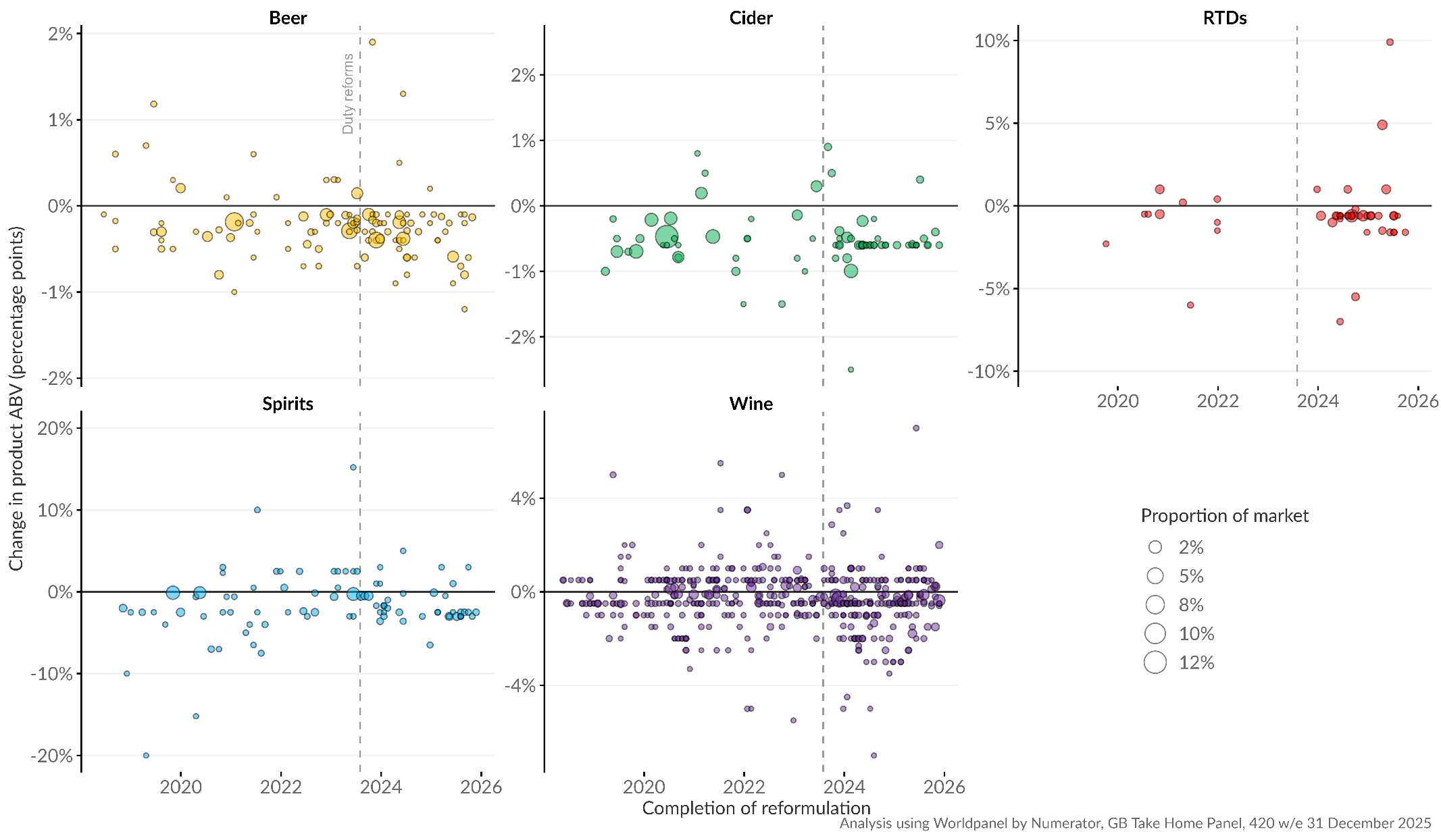


Figure A18 - Timing, magnitude and market share of identified reformulations by beverage type, using a wider tolerance for alcohol-by-volume (ABV) stability


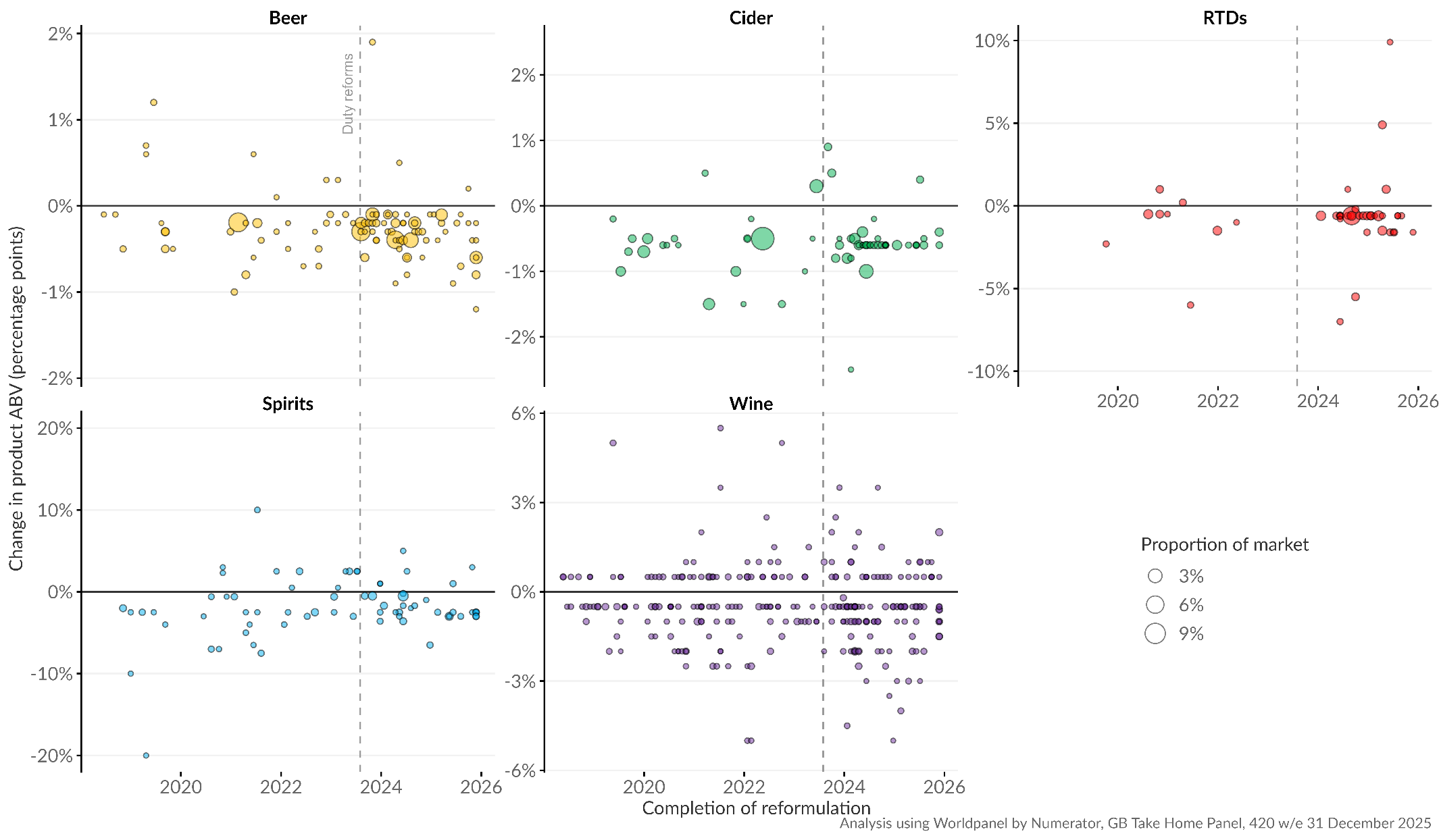


Figure A19 - Timing, magnitude and market share of identified reformulations by beverage type, using a longer period for alcohol-by-volume (ABV) stability


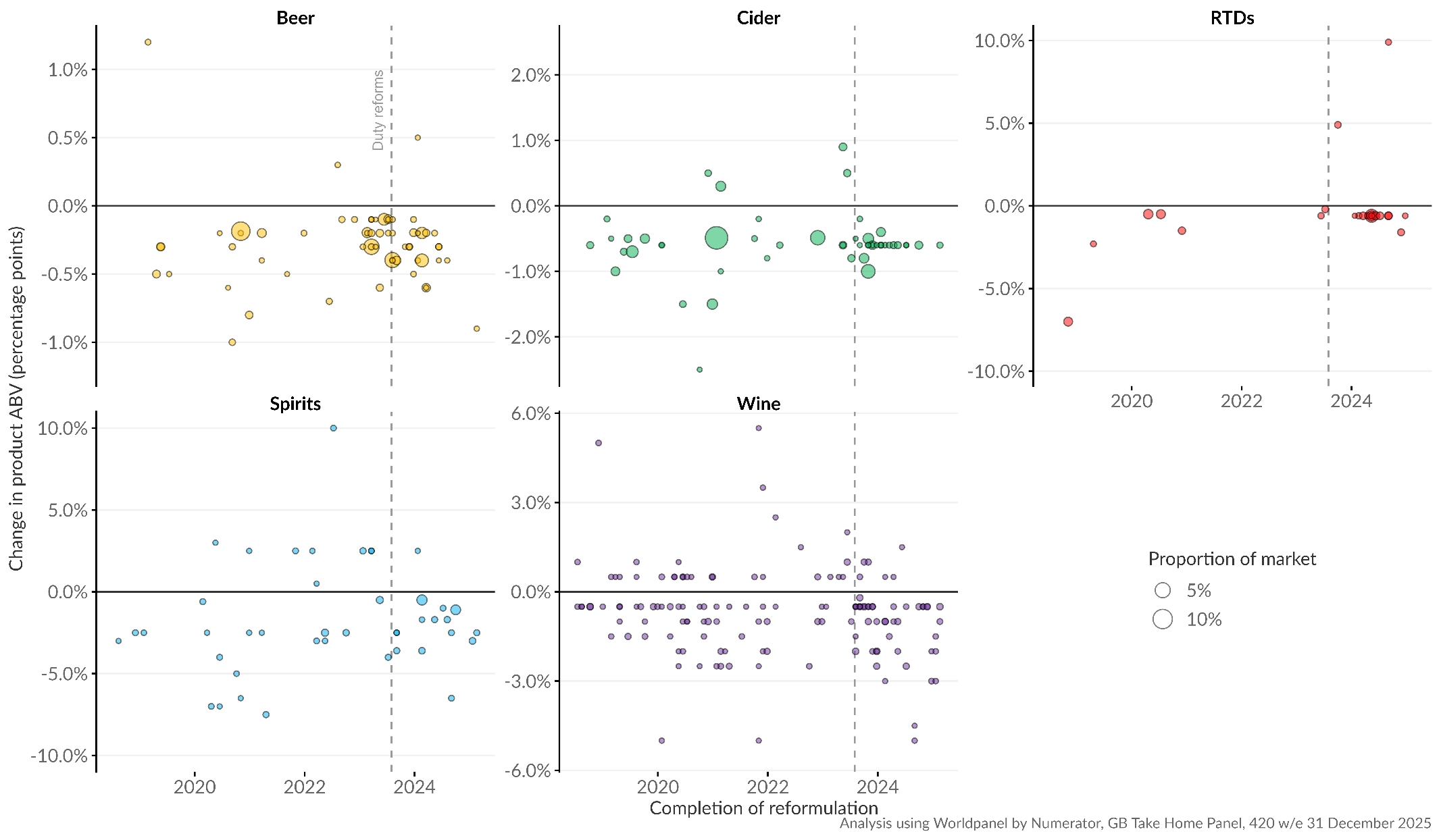

Supplement: Supplementary file 1 — Figure A1. Duty payable per unit of alcohol by product type under the old (panel A) and reformed (panel B) alcohol duty systems. Figure A2. Alcohol duty payable on a 440 mL can of beer under the old and new alcohol duty systems (top panel) and the pre‐reform distribution of beer sales by alcohol‐by‐volume (ABV) (bottom panel). Figure A3. Alcohol duty payable on a 440 mL can of cider under the old and new alcohol duty systems (top panel) and the pre‐reform distribution of cider sales by alcohol‐by‐volume (ABV) (bottom panel). Figure A4. Alcohol duty payable on a 750 mL bottle of wine under the old and new alcohol duty systems (top panel) and the pre‐reform distribution of wine sales by alcohol‐by‐volume (ABV) (bottom panel). Figure A5. Alcohol duty payable on a 700 mL bottle of spirits under the old and new alcohol duty systems (top panel) and the pre‐reform distribution of spirits sales by alcohol‐by‐volume (ABV) (bottom panel). Figure A6. Alcohol duty payable on a 275 mL bottle of Ready‐To‐Drink (RTD) under the old and new alcohol duty systems (top panel) and the pre‐reform distribution of RTD sales by alcohol‐by‐volume (ABV) (bottom panel). Figure A7. Examples of product‐level mean alcohol‐by‐volume (ABV) trajectories, with reformulation periods (highlighted in purple) between windows of ABV stability. Figure A8. Timing and number of identified reformulations by beverage type. Figure A9. Timing, direction, magnitude and market share of all identified reformulations by beverage type. Each bubble represents a reformulation, and the post‐reformulation alcohol‐by‐volume (ABV), while the other end of the line attached to each bubble represents the pre‐reformulation ABV—i.e. a longer line reflects a greater ABV change. Figure A10. Timing, direction, magnitude and market share of all identified wine reformulations. Grey shaded area highlights the 11.5%–14.5% alcohol‐by‐volume (ABV) band affected by the ‘wine easement’. Figure A11. Lower‐strength (< 3.5%) beer market brok [file DAR-45-0-s001.docx]
